# Disruption of VirB6 Paralogs in Anaplasma phagocytophilum Attenuates Its Growth

**DOI:** 10.1128/JB.00301-20

**Published:** 2020-11-04

**Authors:** Francy L. Crosby, Ulrike G. Munderloh, Curtis M. Nelson, Michael J. Herron, Anna M. Lundgren, Yu-Ping Xiao, David R. Allred, Anthony F. Barbet

**Affiliations:** a Department of Infectious Diseases and Immunology, University of Florida, Gainesville, Florida, USA; b Department of Entomology, University of Minnesota, St. Paul, Minnesota, USA; c Emerging Pathogens Institute, University of Florida, Gainesville, Florida, USA; Brigham and Women’s Hospital/Harvard Medical School

**Keywords:** *Anaplasma phagocytophilum*, obligate intracellular pathogens, *Rickettsiales*, tick-borne bacteria, transposon mutagenesis, VirB6, virulence, attenuated growth, type IV secretion

## Abstract

Many pathogenic bacteria translocate virulence factors into their eukaryotic hosts by means of type IV secretion systems (T4SS) spanning the inner and outer membranes. Genes encoding components of these systems have been identified within the order *Rickettsiales* based upon their sequence similarities to other prototypical systems. Anaplasma phagocytophilum strains are obligate intracellular, tick-borne bacteria that are members of this order. The organization of these components at the genomic level was determined in several Anaplasma phagocytophilum strains, showing overall conservation, with the exceptions of the *virB2* and *virB6* genes. The *virB6* loci are characterized by the presence of four *virB6* copies (*virB6-1* through *virB6-4*) arranged in tandem within a gene cluster known as the *sodB-virB* operon. Interestingly, the *virB6-4* gene varies significantly in length among different strains due to extensive tandem repeats at the 3′ end. To gain an understanding of how these enigmatic *virB6* genes function in A. phagocytophilum, we investigated their expression in infected human and tick cells. Our results show that these genes are expressed by A. phagocytophilum replicating in both cell types and that VirB6-3 and VirB6-4 proteins are surface exposed. Analysis of an A. phagocytophilum mutant carrying the Himar1 transposon within the *virB6-4* gene demonstrated that the insertion not only disrupted its expression but also exerted a polar effect on the *sodB-vir*B operon. Moreover, the altered expression of genes within this operon was associated with the attenuated *in vitro* growth of A. phagocytophilum in human and tick cells, indicating the importance of these genes in the physiology of this obligate intracellular bacterium in such different environments.

**IMPORTANCE** Knowledge of the T4SS is derived from model systems, such as Agrobacterium tumefaciens. The structure of the T4SS in *Rickettsiales* differs from the classical arrangement. These differences include missing and duplicated components with structural alterations. Particularly, two sequenced *virB6-4* genes encode unusual C-terminal structural extensions resulting in proteins of 4,322 (GenBank accession number AGR79286.1) and 9,935 (GenBank accession number ANC34101.1) amino acids. To understand how the T4SS is used in A. phagocytophilum, we describe the expression of the *virB6* paralogs and explore their role as the bacteria replicate within its host cell. Conclusions about the importance of these paralogs for colonization of human and tick cells are supported by the deficient phenotype of an A. phagocytophilum mutant isolated from a sequence-defined transposon insertion library.

## Abstract (precis)

Knowledge of the T4SS is derived from model systems, such as Agrobacterium tumefaciens. The structure of the T4SS in *Rickettsiales* differs from the classical arrangement. These differences include missing and duplicated components with structural alterations. Particularly, two sequenced *virB6-4* genes encode unusual C-terminal structural extensions resulting in proteins of 4,322 (GenBank accession number AGR79286.1) and 9,935 (GenBank accession number ANC34101.1) amino acids. To understand how the T4SS is used in A. phagocytophilum, we describe the expression of the *virB6* paralogs and explore their role as the bacteria replicate within its host cell. Conclusions about the importance of these paralogs for colonization of human and tick cells are supported by the deficient phenotype of an A. phagocytophilum mutant isolated from a sequence-defined transposon insertion library.

## INTRODUCTION

The type IV secretion system (T4SS) is a macromolecular protein complex of Gram-negative and -positive bacteria that forms a channel across the cell envelope, allowing the secretion of substrates. Depending on the bacterial species, this multimeric channel is a conduit for direct transfer of DNA, proteins, or effectors into eukaryotic host cells, enabling virulence and survival of bacteria (1–3). In some instances, this system is also involved in DNA uptake from the extracellular milieu (4) or interbacterial killing (5, 6). The T4SS is of major medical relevance, as it is used by several multidrug-resistant bacterial species to spread antibiotic resistance genes (7). The VirB/VirD4 system encoded by the pTi plasmid of Agrobacterium tumefaciens represents the canonical P-T4SS, consisting of 12 subunits, VirB1 throughout VirB11 and VirD4, which serve to deliver oncogenic nucleoprotein particles into plant cells, resulting in the development of crown gall tumors (8).

Anaplasma phagocytophilum (order *Rickettsiales*; family *Anaplasmataceae*) is an obligate intracellular tick-borne bacterium and the causative agent of human granulocytic anaplasmosis (HGA), a notifiable disease since 1998. This pathogen is of increasing concern in the United States and internationally as an emerging agent that causes disease in both humans and animals. Members of this species have a tropism for neutrophils and granulocytes, and infection is characterized by a nonspecific febrile illness with clinical manifestations that range from asymptomatic to fatal disease if left untreated (9–11). Moreover, all members of the order *Rickettsiales* sequenced to date encode components of a T4SS, prototypical to the VirB/VirD system of Agrobacterium tumefaciens (9, 12, 13).

Although the T4SS in A. phagocytophilum superficially resembles that of A. tumefaciens, significant differences exist. For example, in A. tumefaciens the genes that encode the transmembrane channel and coupling proteins are organized in a single locus that contains the *virB* and the *virD* operons (8, 14). However, in A. phagocytophilum these components are distributed in three distal clusters. The first includes *virB8-1*, *virB9-1*, *virB10*, *virB11*, and *virD4*, the second includes *virB2s* and *virB4-2*, and the third comprises *virB3*, *virB4-1*, and four *virB6* paralogs (12, 15). Coinciding with the lack of genes required for peptidoglycan synthesis, A. phagocytophilum lacks *virB1*, a gene than encodes a murein-degrading transglycosylase required for channel assembly across the cell wall (16–19), and *virB5*, which encodes a pilus-associated protein, but has duplicate copies of genes encoding secretion channel-associated proteins (*virB4*, *virB8*, and *virB9* genes), the four copies of *virB6*, and multiple copies (8 to 15) of *virB2*, which vary in number and sequence between A. phagocytophilum strains (15). These differences in organization suggest alternate apparatus assembly, regulation, and substrate transfer (12, 20) and perhaps even assembly of variable surface structures that may modulate host-pathogen interactions such as attachment to different host cells or evasion of host immune responses (21, 22).

Comparative genomics of these components in several A. phagocytophilum strains showed that they are highly conserved among strains, with the exception of *virB2* and *virB6*. The *virB6* loci, the focus of this study, are characterized by the presence of four copies in tandem (*virB6-1* through *virB6-4*) arranged within an operon that contains *sodB*, *virB3*, and *virB4-1*. Interestingly, *virB6-4* varies among strains due to the presence of an extensive repeat domain with various numbers of repeats found at the 3′ end (15). Although VirB6 is essential for substrate secretion and stability of the T4SS (23–26), very little is known about the expression of the four *virB6* paralogs and their role in A. phagocytophilum infection and pathogenesis.

In this study, molecular and biochemical assays led us to conclude that the four *virB6* paralogs are expressed in A. phagocytophilum during *in vitro* infection of human and tick cells and that VirB6-3 and VirB6-4 proteins are surface exposed. Transposon mutagenesis of A. phagocytophilum using the Himar1 system resulted in the isolation of a mutant carrying transposon (Tn) sequences within the *virB6-4* gene. Insertion of Tn sequences within this gene not only disrupted its expression but also had a polar effect on the *sodB-virB* operon. Moreover, altered expression of genes within this operon was linked to attenuated growth of A. phagocytophilum in human and tick cells, indicating the importance of these genes during intracellular replication.

## RESULTS

### The *virB6* loci are transcribed in A. phagocytophilum during infection of human and tick cells.

**FIG 1:**
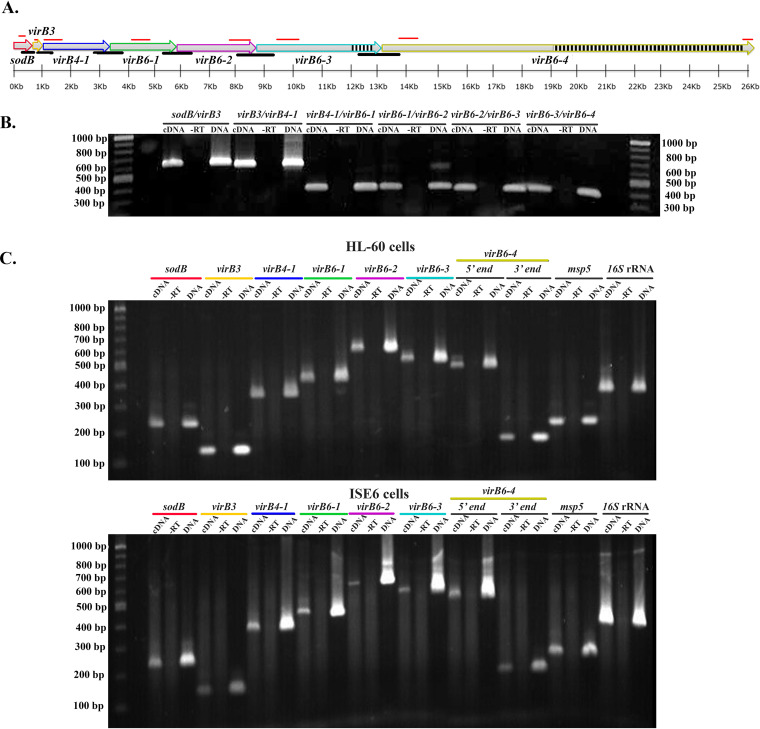
A. phagocytophilum *virB6* paralogs are cotranscribed in mammalian and tick cells. (A) Organization of the *sodB-virB* operon in Anaplasma phagocytophilum. Black lines indicate locations of primers designed to amplify transcripts from intergenic regions *sodB-virB3*, *virB3–virB4-1*, *virB4-1–virB6-1*, *virB6-1–virB6-2*, *virB6-2–virB6-3*, and *virB6-3–virB6-4*. Red lines indicate binding sites for primers designed to amplify transcripts from *sodB*, *virB3*, *virB4-1*, *virB6-1*, *virB6-2*, *virB6-3*, and the 5′ and 3′ ends of *virB6-4*. Black and white boxes depict the repeats region of *virB6-3* and *virB6-4*. The *sodB-virB* operon was drawn to scale using SnapGene software (from GSL Biotech). (B) Agarose gel analysis of cDNA products from *sodB-virB* operon intergenic regions. RT-PCR was performed on total RNA isolated from A. phagocytophilum-infected HL-60 cells. (C) Agarose gel analysis of cDNA products from *sodB* through *virB6-4*. RT-PCR was performed on total RNA isolated from A. phagocytophilum-infected HL-60 and ISE6 cells. Genomic DNA was used as a positive control, and reactions without reverse transcriptase (-RT) were used as negative controls. A 100-bp/1-kb DNA ladder was used. The *msp5* and 16S rRNA genes were used as internal controls to ensure integrity of RNA/cDNA. Data were obtained from two independent experiments.

The *virB6* loci are organized within an operon structure (*sodB-virBs*) comprised of *sodB*, *virB3*, *virB4-1*, *virB6-1*, *virB6-2*, *virB6-3*, and *virB6-4* in tandem (Fig. 1A). Prior work failed to clarify the polycistronic nature or transcription status of each of these genes (15, 27, 28). Therefore, to help us understand the contribution of this locus to A. phagocytophilum virulence, we determined the cistronic organization and transcription of the *sodB-virB*s locus in both human and tick cells. Moreover, since the peptide used to raise antibodies against VirB6-4 represents sequences from the C-terminal repeat region, we also queried whether the full length of this gene is transcribed in both cell types by targeting transcripts from the 5′ and 3′ ends. Total RNA isolated from A. phagocytophilum-infected cells was reverse transcribed with random hexamer primers and the cDNA template used for PCR amplification with primers targeting sequences from intergenic regions and each gene within the operon (Fig. 1A). Reverse transcription-PCR (RT-PCR) products of appropriate size from the intergenic regions between *sodB-virB3*, *virB3*–*virB4-1*, *virB4-1*–*virB6-1*, *virB6-1*–*virB6-2*, *virB6-2*–*virB6-3*, and *virB6-3*–*virB6-4* genes were detected (Fig. 1B), indicating that these genes are polycistronically transcribed. Moreover, transcripts from each gene and full-length *virB6-4* were detected in both HL-60 and tick ISE6 cells (Fig. 1C).

### The four VirB6 paralogs were detected in A. phagocytophilum-infected human and tick cells.

The prototypical A. tumefaciens VirB6 is an integral membrane protein of ∼300 amino acid residues and four to five transmembrane domains (TMD1 through TMD5) (26, 29). Comparison of VirB6 protein sequences from A. tumefaciens with paralogs from A. phagocytophilum, Anaplasma marginale and Ehrlichia chaffeensis indicate sequence conservation restricted only toward the A. tumefaciens central DNA-transferring TrbL/VirB6 domain, specifically, three central TMDs and a cytoplasmic loop found between TMD3 and TMD4 (see Fig. S1A in the supplemental material). In A. tumefaciens, a tryptophan residue within the central cytoplasmic loop is essential for localization of this protein at the cell pole (30). This residue appears to be conserved among the VirB6 protein family (26, 30), including the five VirB6 paralogs from *Rickettsia* species (20, 31). Likewise, we found this residue to be invariant in VirB6 proteins from A. phagocytophilum, A. marginale, and *E. chaffeensis* (Fig. S1A), indicating conservation of this residue in *Rickettsiales*. Relative to A. tumefaciens, and similar to other *Rickettsiales* (12, 32), A. phagocytophilum encodes much larger VirB6 proteins characterized by extended N- or C-terminal hydrophilic regions flanking the TrbL/VirB6 domain (Fig. S1B), which place them within the “extended VirB6-like protein” subtype (4, 14, 16). *In silico* topology analysis indicated that in A. phagocytophilum, VirB6-1 and VirB6-2 have nine and seven TMDs, respectively, and a putative amino-terminal cleavable signal peptide (SP) (Fig. S1B). However, it is possible that SP residues from these proteins correspond to the first TMD, as it is known that prediction algorithms often confound TMDs with the SP due to similar biochemical characteristics (33). On the other hand, VirB6-3 and VirB6-4 have eight and six TMDs, respectively, and are predicted to be lipoproteins, as both contain highly conserved lipobox sequences (Fig. S1B). Common to these paralogs is the clustering of TMDs toward the TrbL/VirB6 hydrophobic region. VirB6-1, VirB6-2, and VirB6-3 carry the TrbL/VirB6 domain located toward the C terminus, while in VirB6-4 this domain is found toward the N terminus (Fig. S1B).

To determine expression of *virB6* paralogs as proteins, specific antibodies against A. phagocytophilum strain HZ VirB6 proteins were prepared using synthetic peptides derived from unique predicted antigenic epitopes of each protein (Fig. S1B and Table 1). An enzyme-linked immunosorbent assay (ELISA) was performed to determine the titer of rabbit polyclonal antibodies specific to VirB6-1, -6-2, -6-3, and -6-4 peptides using conjugates of the VirB6 peptides with ovalbumin or ovalbumin alone as a coating agent. This assay showed that immunized animals developed high antibody titers against the peptides. In addition, there was a lack of reactivity of the preimmunization serum against the peptide (Fig. S2).

**TABLE 1:** Peptides used to generate polyclonal antibodies against VirB6 proteins

Peptide	Sequence	Length (residues)
VirB6-1	C-SNYAWPKNRVSSSRYIEVCYRHPLGTVYMSPYVAARLGFAGRDPKEVLKESKYPRE	56
VirB6-2	C-YPLYFSSKLGGGSGYYNSWYKVAKGEAPYITDGIPEYLHISGGIVKPSSMEKLDDEHE	58
VirB6-3	C-REYRPGDGGVGEGDDRVYGSTTAGSGISGDAAGIDARGDHAREHQPADVGVGEGADRVSG	60
VirB6-4	C-TELPREVVPEATEYGTKPDDQDGDKGDLRPERLDPDIGDGSAIEDEVEVRSSRSSESTDSVPSEVTERDA	70

**FIG 2:**
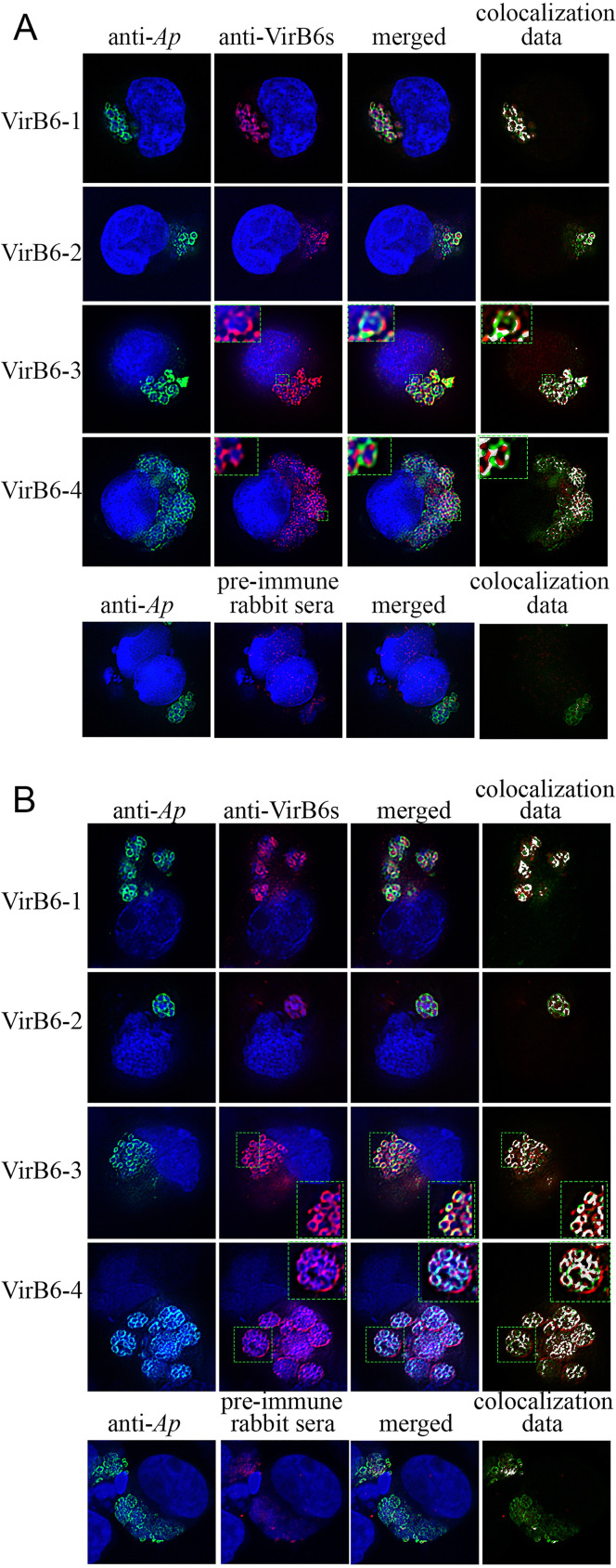
A. phagocytophilum (*Ap*) VirB6 proteins are synthesized in infected HL-60 and ISE6 cells. Shown are representative dual immunofluorescence images of HL-60 (A) and ISE6 (B) cells infected with A. phagocytophilum. To indicate immunolabeling of VirB6-3 and VirB6-4, boxes delineated by the dotted lines are magnified in the insets. Infected cells were fixed and viewed by indirect immunofluorescence microscopy to determine immunoreactivity with A. phagocytophilum-infected mouse sera (green) and anti-VirB6-1, -VirB6-2, -VirB6-3, and -VirB6-4 sera (red). Host cell nuclei and bacterial DNA were stained with DAPI (blue). Preimmune rabbit serum was used as a negative control. The RG2B Colocalization ImageJ plug-in (C. P. Mauer, Northwestern University) was used to assess the overlap between the green and red channels. Representative images from two independent experiments are shown.

The expression of the individual VirB6 proteins in infected HL-60 and ISE6 cells was evaluated by immunofluorescence microscopy. As a localization control, antisera from mice infected with A. phagocytophilum strain HZ (with antibody responses mainly targeting the dominant surface protein MSP2/P44) (34, 35) were used. Binding of the infection sera to A. phagocytophilum generated a green fluorescent signal matching the known location of MSP2/P44 on the periphery of bacteria (Fig. 2). This allowed us to confidently identify the specific localization of rabbit anti-VirB6 peptides to A. phagocytophilum. Dual fluorescence indicated that the red fluorescent signals from VirB6-1, VirB6-2, VirB6-3, and VirB6-4 epitopes were specific to A. phagocytophilum organisms in infected HL-60 and ISE6 cells as shown by superimposed images and colocalization data (Fig. 2). Similar binding was not observed in infected cells reacted with preimmune rabbit sera. Interestingly, VirB6-3 and VirB6-4 appeared to be located predominantly at the periphery of A. phagocytophilum as multiple foci or punctate structures when growing in HL60 cells (Fig. 2A). In ISE6 cells, the red fluorescent signals from VirB6-3 and VirB6-4 were also located at the periphery of bacteria (Fig. 2B). Conversely, the signal from VirB6-4 was additionally observed in a location consistent with it being associated with the parasitophorous vacuole (PV), and the originating signal did not overlap the green fluorescence as indicated by colocalization data (Fig. 2B). However, as we did not use antibodies specific to PV associated proteins, we could not determine this localization unambiguously.

**FIG 3:**
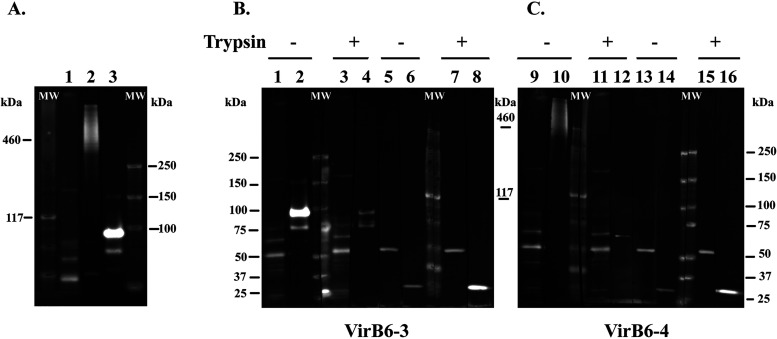
VirB6-3 and VirB6-4 proteins are located on the surface of A. phagocytophilum. (A) Proteins from A. phagocytophilum isolated from HL-60 were separated by Tris acetate polyacrylamide gel electrophoresis. For immunoblotting, proteins were transferred to PVDF membranes and reacted with rabbit preimmune serum (lane 1) and rabbit anti-VirB6-4 or anti-VirB6-3 serum (lanes 2 and 3). (B and C) Intact A. phagocytophilum organisms were incubated with (+) or without (−) trypsin and solubilized, and equal amounts of proteins were separated by Tris acetate polyacrylamide gel electrophoresis. PVDF membranes with transferred proteins were processed for detection. (B) Rabbit anti-VirB6-3 serum (lanes 2 and 4), rabbit preimmune serum (lanes 1 and 3), normal mouse serum (NMS) (lanes 5 and 7), and polyclonal mouse anti-mCherry antibodies (lanes 6 and 8); (C) rabbit anti-VirB6-4 serum (lanes 10 and 12), rabbit preimmune serum (lanes 9 and 11), NMS (lanes 13 and 15), and polyclonal mouse anti-mCherry antibodies (lanes 14 and 16). “MW” indicates protein standards. Immunoblots are representative of those from three independent experiments.

Due to the C-terminal hydrophilic repeat domains of VirB6-3 and VirB6-4, the focus of this work, it has been proposed that these proteins or their C-terminal domains are surface exposed or proteolytically released into the host cell (15). In addition, evidence of surface exposure of the extended VirB6 proteins has been presented for other *Rickettsiales* (36–38). Hence, we confirmed their expression in infected HL-60 cells by Western immunoblotting and determined whether these proteins are surface exposed by trypsin digestion of intact A. phagocytophilum organisms. Western immunoblotting showed specific protein bands in samples reacted with sera from VirB6-3 and VirB6-4 peptide-immunized rabbits that were not detected in samples reacted with preimmune sera (Fig. 3A). The sizes of the bands detected matched the predicted molecular weights of VirB6-3 (158.3 kDa) and VirB6-4 (predicted mass of 470 kDa, but can migrate anomalously because of larger repeat sequences). Additional protein bands of smaller size than predicted were detected in the sample reacted with sera against VirB6-3. It is possible that this protein undergoes proteolytic posttranslational processing that results in smaller molecules or represents nonspecific degradation products (Fig. 3A).

To confirm that VirB6-3 and VirB6-4 are surface-exposed proteins in A. phagocytophilum, we performed trypsin digestion of intact A. phagocytophilum organisms. For these experiments, we used mCherry-tagged A. phagocytophilum organisms that carry the Himar1 Tn integrated within an intergenic region between the HGE1_00520 (phosphatidate cytidylyltransferase) and the HGE1_00525 (*p44*-66) genes and whose growth in HL-60 and ISE6 cells is comparabe to that of the parental strain A. phagocytophilum strain HGE1 (39). Intact A. phagocytophilum organisms were incubated with trypsin, followed by solubilization and Western blotting using rabbit anti-VirB6-3 and anti-VirB6-4 sera. Mouse anti-mCherry antibody was used as a control to show that trypsin would not breach the cell wall, as this protein localizes to the bacterial cytosol and is not surface exposed. Treatment of bacteria with trypsin completely digested the protein band of 158 kDa and dramatically reduced the detection of smaller protein bands in samples reacted with anti-VirB6-3 antisera relative to untreated samples (Fig. 3B). Likewise, anti-VirB6-4 sera did not detect VirB6-4 protein in samples digested with trypsin relative to untreated controls (Fig. 3C). In trypsin-treated samples, mCherry was not reduced or diminished in size, indicating that reduction of VirB6-3 and VirB6-4 was due to trypsin treatment of the cell surface and not to damaged organisms.

### Isolation of a VirB6-4 mutant generated by transposon mutagenesis.

Anaplasma phagocytophilum strain HGE1, subsequently referred to here as the wild type (wt), was subjected to Himar1 Tn mutagenesis (40, 41). Tn sequencing (Tn-seq) using the Illumina platform detected a Tn insertion into the *virB6-4* open reading frame. Cloning by limiting dilution allowed the isolation of a population of organisms isogenic for the Tn insertion within this gene. Therefore, these bacteria are designated here as *virB6-4*::Himar1 mutants.

**FIG 4:**
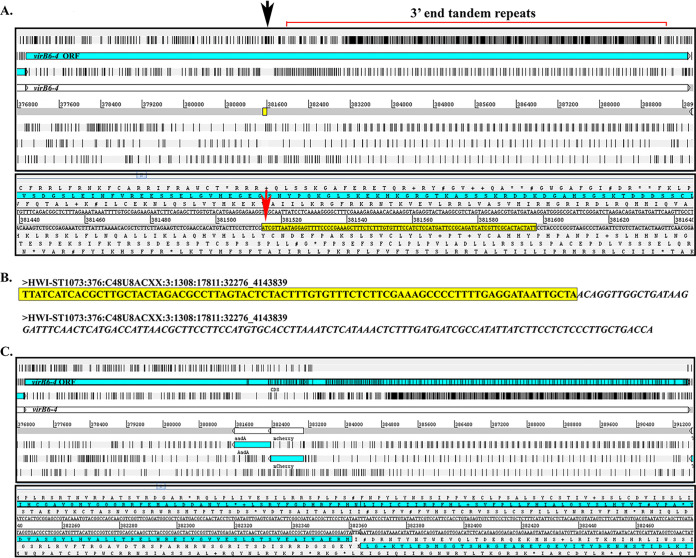
Himar1 Tn insertion site in the A. phagocytophilum *virB6-4* gene. (A) Artemis (genome browser and annotation tool; Wellcome Sanger Institute, UK) window showing A. phagocytophilum wild-type *virB6-4* gene used as a reference for the location of the Himar1 insertion site. The first panel shows a “zoomed out” view of the *virB6-4* DNA sequence translated into six reading frames with black bars indicating stop codons. The open reading frame (ORF) for *virB6-4* is shown in light blue. The black arrow indicates the Himar1 insertion location, 5′ upstream the 3′ end tandem repeats. The yellow box is “zoomed in” in the panel below to show in detail the *virB6-4* sequences flanking the Himar1 Tn (yellow highlight), including the TA dinucleotide site required for successful transposition (red arrow). (B) Paired-end DNA sequencing reads indicating the same A. phagocytophilum sequences as described above (yellow highlight) and Himar1 Tn sequences (italics). (C) Artemis window of *in silico* cloning of the Himar1 Tn in the opposite orientation to *virB6-4*. Black bars within the *virB6-4* ORF indicate putative stop codons.

Although the genome sequence of A. phagocytophilum strain HGE1 (GenBank accession number APHH00000000) is available, the 3′ end repeat region of *virB6-4* is unresolved, containing gaps. Therefore, for our analysis we used the complete *virB6-4* sequence from the A. phagocytophilum strain HGE1 mutant DU1 (GenBank accession number NZ_LASP01000002) as a reference. Alignment of Illumina reads to the reference genome mapped the Tn insertion at the TA dinucleotide coordinates (381514..381515) 5′ upstream from the extensive tandem repeat domain at the 3′ end (Fig. 4A and B). This analysis indicated that the *mCherry* and *aadA* genes from the Tn are integrated in the opposite orientation to *virB6-4* (Fig. 4C). Orientation of the Himar1 transposon was also confirmed by Sanger sequencing.

### Himar1 Tn insertion within *virB6-4* abolished its expression and had a polar effect on the *sodB-virB*s operon.

**FIG 5:**
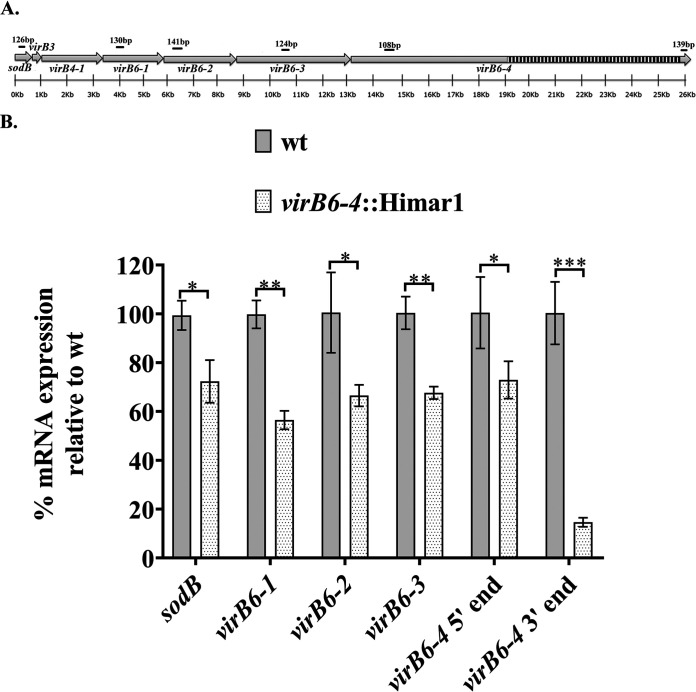
Deletion of *virB6-4* affects transcription of upstream genes. (A) Binding sites of primers designed to target transcripts from *sodB*, *virB6-1*, *virB6-2*, *virB6-3*, and the 5′ and 3′ ends of *virB6-4*. (B) Changes in expression of these genes were calculated based on geometric mean of the threshold cycle (Δ*C_T_*) values from reference genes, and the results were expressed as percentage of expression, with a 100% expression level being assigned to the control group, in this case, wt A. phagocytophilum. Transcripts from the *rpoB* and *groEL* genes were used as reference for data normalization. Bar lengths represent the percentage of expression of *sodB*, *virB6-1*, *virB6-2*, *virB6-3*, and the 5′ and 3′ ends of *virB6-4* in A. phagocytophilum (wt) and the *virB6-4*::Himar1 mutant. *, *P = *0.01 to 0.05; **, *P = *0.001 to 0.01; ***, *P < *0.001. Data were obtained from three independent experiments with two technical replicates.

*In silico* cloning of Tn sequences within the *virB6-4* gene suggested that its insertion resulted in a shift in the reading frame that was predicted to lead to premature termination of translation and the expression of a truncated protein (Fig. 4C). To test this assumption and determine whether the inserted transposon exerted polar effects on the expression of adjacent genes, we examined the expression of *virB6-4* and upstream genes. Reverse transcription-quantitative PCR (RT-qPCR) was used to quantitatively determine differences in the expression of gene components of the *sodB-virB* operon in the A. phagocytophilum wt versus the *virB6-4*::Himar1 mutant. For this, total RNA from HL-60 cells infected with the A. phagocytophilum wt or the *virB6-4*::Himar1 mutant was reverse transcribed using random hexamer primers. The resulting cDNAs were quantified by real-time PCR amplification using primers targeting *sodB*, *virB6-1*, *virB6-2*, *virB6-3*, and the 3′ and 5′ ends of *virB6-4* (Fig. 5A). Relative expression levels of these genes were normalized to the geometric mean of the reference genes *rpoB* and *groEL*. RT-qPCR analysis revealed significantly reduced transcripts from *sodB* (mean ± standard deviation [SD], 69.3% ± 6.83%), *virB6-1* (54.26% ± 3.67%), *virB6-2* (63.96% ± 5.8%), *virB6-3* (64.97% ± 3.40%), the *virB6-4* 5′ end (70.14% ± 9.07%), and the *virB6-4* 3′ end (14.03% ± 1.49%) in the *virB6-4*::Himar1 mutant relative to the values for the wt, which were as follows: *sodB*, 99.38% ± 6.02%; *virB6-1*, 99.79% ± 5.71%; *virB6-2*, 100% ± 16.45%; *virB6-3*, 100% ± 6.67%; the *virB6-4* 5′ end, 100% ± 14.65%; the *virB6-4* 3′ end, 100% ± 12.79% (Fig. 5B). Initially, for gene expression data normalization, we also evaluated transcripts from the *msp5* gene. However, during this analysis, we observed decreased transcription of this gene in the *virB6-4*::Himar1 mutant (mean ± SD, 78.44% ± 11.66%) relative to wt bacteria (99.91% ± 3.41%) (Fig. S3), suggesting that Himar1 insertion within *virB6-4* may also have *trans*-acting effects on the expression of additional, more distant genes.

**FIG 6:**
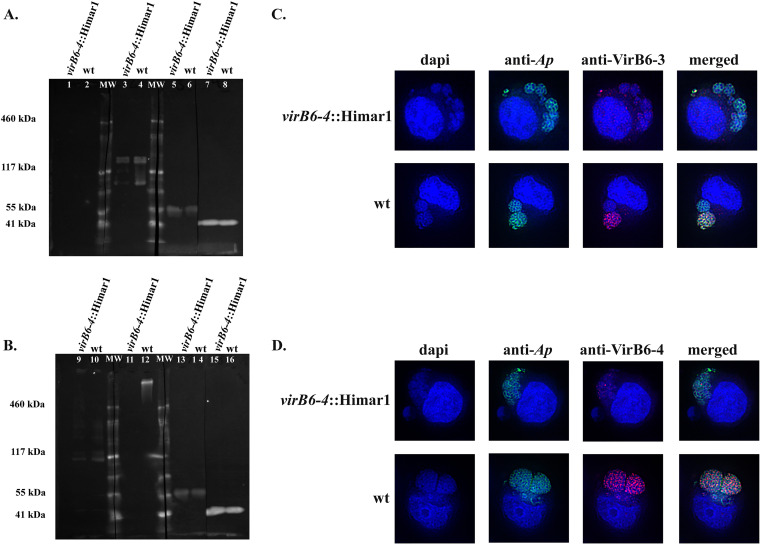
Synthesis of VirB6-4 and VirB6-3 is disrupted in the *virB6-4*::Himar1 mutant. Equal amounts of proteins from host cell purified *virB6-4*::Himar1 and wt A. phagocytophilum were separated by Tris acetate polyacrylamide gel electrophoresis. (A) PVDF membranes of transferred proteins were reacted with rabbit preimmune serum (lanes 1 and 2), rabbit anti-VirB6-3 serum (lanes 3 and 4), and dog preimmune serum (lanes 5 and 6). Polyclonal dog serum infected with A. phagocytophilum, with immune responses mainly targeting the MSP2/P44 surface protein, was used as a loading control to indicate equal loading of proteins from the *virB6-4*::Himar1 mutant and wt A. phagocytophilum (lanes 7 and 8). (B) PVDF membranes of transferred proteins were reacted with rabbit preimmune serum (lanes 9 and 10), rabbit anti-VirB6-4 serum (lanes 11 and 12), dog preimmune serum (lanes 13 and 14), and A. phagocytophilum-infected dog serum (loading control; lanes 15 and 16). (C and D) HL-60 cells infected with A. phagocytophilum *virB6-4*::Himar1 or the wt were fixed and viewed by indirect immunofluorescence microscopy to determine immunoreactivity with A. phagocytophilum-infected mouse serum (green) and rabbit anti-VirB6-3 (C, red) or anti-VirB6-4 (D, red) serum. Representative immunoblots were obtained from three independent experiments. Representative immunofluorescence assay images from several micrographs are shown.

To determine whether decreased mRNA levels from the *virB6-3* and *virB6-4* genes in the *virB6-4*::Himar1 mutant correlate with altered VirB6-3 and VirB6-4 protein levels, we performed Western blot analysis using polyclonal rabbit anti-VirB6-3 and anti-VirB6-4 sera. To be sure that observed changes in VirB6-3 and VirB6-4 protein levels in the *virB6-4*::Himar1 mutant relative to wt bacteria are due to altered protein expression rather than differences in sample loading, we used polyclonal dog serum against A. phagocytophilum, with antibody responses mainly targeting the dominant surface protein MSP2/P44 (42 to 44 kDa), as a loading control. Western blotting indicated a reduction in VirB6-3 expression in the *virB6-4*::Himar1 mutant compared to the wt (Fig. 6A). The ratio of the amount of VirB6-3 in the *virB6-4*::Himar1 mutant relative to the VirB6-3 amounts in the wt was determined by densitometry, normalized to the signal obtained from the MSP2/P44 band and converted to percentage. This indicated decreased levels of VirB6-3 expression in the *virB6-4*::Himar1 mutant (mean ± SD, 24.27% ± 14.82%) relative to the wt (100% ± 14.43%). VirB6-4 protein was detected only in protein samples from wt organisms and not in the *virB6-4*::Himar1 mutant (Fig. 6B). Bands of sizes corresponding to VirB6-3 and VirB6-4 proteins were not observed in samples reacted with the negative-control rabbit preimmune sera (Fig. 6A and B). Consistent with Western blot analysis, dual immunofluorescence of HL-60 cells infected with the *virB6-4*::Himar1 or wt strain indicated a dramatic reduction in the signals from VirB6-3 epitopes in the *virB6-4*::Himar1 mutant-infected relative to wt-infected cells (Fig. 6C). Likewise, fluorescent signals from VirB6-4 epitopes in cells infected with the *virB6-4*::Himar1 mutant were virtually absent compared to those in wt-infected cells (Fig. 6D).

### The *virB6-4*::Himar1 mutant has a reduced proliferation *in vitro*.

**FIG 7:**
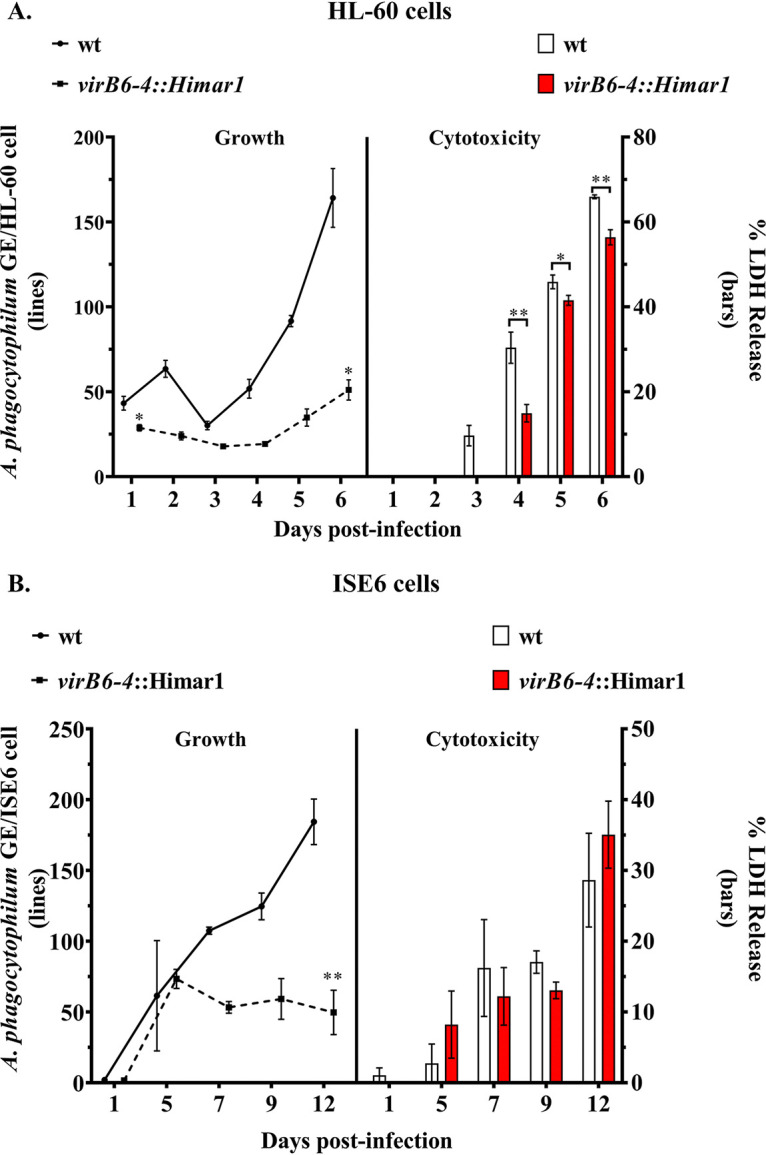
Growth and cytotoxicity of the *virB6-4*::Himar1 mutant. The growth of A. phagocytophilum *virB6-4*::Himar1 (dotted line) versus wild-type (wt) (black line) in infected HL-60 (A) and ISE6 (B) cells was followed by determining the number of A. phagocytophilum genome equivalents per host cell targeting the A. phagocytophilum single-copy gene *msp5* and the *tlr9* or *crt* genes from HL-60 and ISE6 cells, respectively. Bars indicate A. phagocytophilum *virB6-4*::Himar1 and wild-type cytotoxicity to host cells as indicated by the levels of LDH release into the cell culture supernatants. The culture medium was not changed at any time during the course of this experiment. *, *P = *0.01 to 0.05; **, *P = *0.001 to 0.01. Data were obtained from three independent experiments with three technical replicates.

We further evaluated the growth kinetics of the *virB6-4*::Himar1 mutant versus the wt by determining the numbers of A. phagocytophilum genome equivalents per cell (ApGE/cell) using duplex qPCR. For this, we used specific primers and probes targeting the A. phagocytophilum *msp5* gene and the Toll-like receptor 9 (*tlr9*) gene in HL-60 cells or the calreticulin gene (*crt*) in ISE6 cells. During the course of infection in HL-60 cells, the *virB6-4*::Himar1 mutant maintained a reduced growth, as evidenced by significantly lower numbers of ApGE/cell (mean ± standard error of the mean [SEM], 28.82 ± 1.870 [*P* = 0.0317] at day 1 postinfection [p.i.] and 51.11 ± 5.966 [*P* = 0.0035] at day 6 p.i.) relative to ApGE/cell in cells infected with wt bacteria at the same time points (43.26 ± 4.05 and 164.2 ± 17.28 on days 1 and 6 p.i., respectively) (Fig. 7A). On the other hand, during the first 3 days p.i., there were no significant differences in the numbers of ApGE/cell in ISE6 cells infected with the *virB6-4*::Himar1 mutant relative to the wt. However, after day 3 p.i., the growth of the mutant declined, and by day 12 p.i., the numbers of ApGE/cell were significantly lower (mean ± SEM, 49.68 ± 15.6) (*P = *0.0039) than in cells infected with wt bacteria (184.35 ± 4.43) (Fig. 7B).

Given the reduced growth exhibited by the *virB6-4*::Himar1 mutant, we expected decreased levels of host cell death. To determine this, aliquots of cell culture supernatants collected from *virB6-4*::Himar1- or wt-infected HL-60 and ISE6 cells were used to measure the levels of host cell lactate dehydrogenase (LDH cytotoxicity assay) released into the cell medium due to the pathogenic effects exerted by the bacteria. Effectively, the reduced burden of *virB6-4*::Himar1 organisms in HL-60 cells was associated with decreased host cell death, as there was a significantly reduced level of LDH in supernatants from *virB6-4*::Himar1-infected HL-60 cells relative to wt-infected cells at all tested times p.i. (Fig. 7A). In contrast, the reduced infection of ISE6 cells with the *virB6-4*::Himar1 mutant did not correlate with decreased host cell death, as the levels of LDH released in supernatants from infected cells were not significantly different from those in wt-infected cells at all tested times p.i. (Fig. 7B).

## DISCUSSION

The role of the A. phagocytophilum T4SS during infection of human host and tick vector cells remains unclear. Here, we show that all four *virB6* paralogs of A. phagocytophilum are cotranscribed into a polycistronic message that includes *sodB*, *virB3*, and *virB4-1* during *in vitro* infection of mammalian and tick cells. VirB6-1, VirB6-2, VirB6-3, and, strikingly, a large VirB6-4 protein (∼470 kDa) were also detected in infected cultures. Given the unusual C-terminal structural extensions of VirB6-3 and VirB6-4 in A. phagocytophilum, we focused most of our work on characterization of the expression and potential subcellular localization of these two proteins. Evidence obtained from this work suggests that these proteins have evolved to serve a fundamentally different function in *Rickettsiales* than in A. tumefaciens. For example, dual labeling of A. phagocytophilum suggests that VirB6-3 and VirB6-4 are distributed throughout the bacterial cell membrane in infected HL-60 and ISE6 cells, whereas in A. tumefaciens, VirB6 is localized mainly to a single pole (30).

Surface trypsin digestion of host cell-free intact A. phagocytophilum provided evidence that major C-terminal domains of VirB6-3 and VirB6-4 are surface exposed. This also contrasts with A. tumefaciens, in which VirB6 consists of a periplasmic N terminus, four or five transmembrane domains, and a cytoplasmic C terminus (26, 29). Topology similar to that of A. tumefaciens was also reported for VirB6 in *Brucella* (42) and ComB6 (VirB6 homolog) in H. pylori (43). Although further experimental evidence is required, surface trypsin digestion data suggest that the C termini of A. phagocytophilum VirB6-3 and VirB6-4 are surface exposed on the outer membrane, as the peptide sequences used for the preparation of antibodies were derived from VirB6-3 and VirB6-4 C-terminal repeat sequences. It has been proposed that the extended C terminus may be cleaved and released into the target cell to effect regulatory functions (4, 15). This localization would optimize such a transfer. Hence, these data provide initial experimental evidence for an extracytoplasmic extended C terminus in polytopic “extended VirB6-like proteins” in bacteria of the P-T4SS group. Extended C-terminal domains have been found on the F-T4SS of Escherichia coli (TraG) and SXT-ICE (TraGSXT) of Vibrio cholerae. These C-terminal hydrophilic domains function during mating pair formation in association with TraS protein that is found in the inner membrane of the donor of the pair in a process known as entry exclusion to block transfer of redundant DNA (14).

Conforming to the case with other *Rickettsiales* (20), bioinformatics analysis indicated that sequence conservation of A. phagocytophilum VirB6 paralogs relative to VirB6 from A. tumefaciens is restricted to the TMDs and a central cytoplasmic loop. In addition, this analysis predicted SP sequences for VirB6-1 and VirB6-2 and identified VirB6-3 and VirB6-4 as putative lipoproteins given the presence of conserved lipobox signal sequences. Despite the importance of lipoproteins in physiology, virulence, and host immune evasion, very little is known about their role in A. phagocytophilum. In other *Rickettsiales*, such as *E. chaffeensis* (44) and the *Wolbachia* symbiont of filarial nematodes (38), VirB6-3, but not VirB6-4, has also been identified as a putative lipoprotein. Moreover, antibodies reacting with VirB6-3 proteins from *E. chaffeensis* and *Wolbachia* species were detected in sera from dogs infected with *E. chaffeensis* (44) and in sera from humans infected with the filarial nematode Wuchereria bancrofti (38). This is additional evidence that in *Rickettsiales* this protein not only is surface exposed but also induces immune responses. We noted that A. phagocytophilum contains genes that encode proteins required for lipoprotein biosynthesis, such as the prolipoprotein diacylglyceryl transferase (Lgt; GenBank accession number AGR79154) and the lipoprotein signal peptidase (LspA; GenBank accession number AGR79052.1) but not the apolipoprotein *N*-acyltransferase (Lnt/CutE). It is known that *Wolbachia* (45) synthesizes only diacyl-lipoproteins, which are involved in triggering of inflammatory responses (46). The fact that VirB6-3 and VirB6-4 are surface proteins suggests that they could be targeted as potential vaccine candidates against closely related A. phagocytophilum strains.

Transposon mutagenesis using the Himar1 system followed by high-throughput genome sequencing to identify Tn insertion loci in these transformants identified a population of mutants carrying the transposon sequences within the coding region of the *virB6-4* gene. Relative gene expression experiments demonstrated that insertion of transposon sequences into *virB6-4* not only resulted in the loss of its expression but also altered the expression of upstream genes, including *sodB*, *virB6-1*, *virB6-2*, and *virB6-3*, suggesting that gene expression from the entire operon (including *virB3* and *virB4-1*) was disrupted. As the transposon sense strand is found in the opposite orientation to *virB6-4*, it is possible that transcriptional readthrough beyond Tn sequences generated antisense transcripts that reduced the expression of sequences upstream of *virB6-4*. Similar outcomes obtained by Himar1 insertion have been observed in several bacterial species, including tick-borne bacteria (47–49). Consistent with gene expression data, Western blotting and immunofluorescence indicated the absence of VirB6-4 and decreased levels of VirB6-3 in the *virB6-4*::Himar1 mutant relative to wt A. phagocytophilum. Importantly, our data show that the disruption of *virB6-4* and the altered expression of upstream genes significantly decreased A. phagocytophilum infection in human and tick cells *in vitro*, indicating the important role of these T4SS components in the intracellular survival of A. phagocytophilum in these two different environments. Further work is required to determine whether reduced infection is due to a decreased replication or failure to evade host killing mechanisms such as avoidance of lysosomal degradation or failure to escape oxidative damage (50).

Given the polar effect exerted by the Himar1 insertion within the *virB6-4* gene, it is not possible to assign the mutant phenotype to this gene alone, as expression of the other *virB6* paralogs *virB3*, *virB4*-1, and *sodB* was also affected. However, it is possible that the altered expression levels of T4SS components present in this operon affect the abundance of other T4SS components, impairing the assembly and function of the secretion system and resulting in poor growth. A similar effect has been observed in other bacterial species, such as A. tumefaciens and a *Brucella* sp., carrying mutations in T4SS components, specifically *virB6* mutations (25, 51). In *Brucella* species, VirB3 and VirB6 are essential for persistence in mice (25). Another important consequence of disruption of *virB6-4* relates to the observed reduced expression of *sodB* in the *virB6-4*::Himar1 mutant as shown here. Expression of superoxide dismutase (SOD) in pathogenic bacteria contributes to virulence, as it helps them to escape killing by reactive oxygen species released by the host defense mechanisms (52, 53). In that context, it is worth noting that the *virB6-4*::Himar1 mutant showed reduced expression of *sodB*. It is known that A. phagocytophilum inhibits NADPH oxidase, thus avoiding killing by toxic oxygen intermediates (50, 54–57). However, it is not known which A. phagocytophilum effector proteins participate in this inhibitory process. It is possible that A. phagocytophilum uses SOD to protect itself from oxidative damage, and reduced expression of this protein as shown for the *virB6-4*::Himar1 mutant may contribute to impaired proliferation in infected cells.

The isolation of the *virB6-4*::Himar1 mutant with an altered expression pattern in the *sodB-virB* operon supports the contribution of these proteins to A. phagocytophilum infection. Reduced transcript levels from *msp5* in the *virB6-4*::Himar1 mutant may reflect not only modification or remodeling of membrane protein components but also that genes from the *sodB-virBs* operon are possibly linked to regulation of additional genes. Therefore, comparative proteomics of *virB6-4*::Himar1 mutant and wt A. phagocytophilum will be of great significance to define differentially expressed proteins and pathways associated with the slow-growth phenotype of this mutant. Additionally, expression of a defective T4SS not only will help to understand how the T4SS functions but also will aid in the identification of T4SS substrates and will clarify the role of this operon in persistent infection.

The order *Rickettsiales* includes pathogens of high importance in human and veterinary health and, potentially, bioterrorism. Despite their genetic diversity, the different cellular niches they occupy, and the variety of hosts they infect, an element common to members of this order is the synteny of the *virB3*, *virB4*, and *virB6* paralogs (4 copies *in Anaplasmataceae* and 5 copies in *Rickettsiaceae*) that are arranged in tandem within an operon unit (12). This genomic organization is most likely due to functional constrains. Hence, results obtained in working with A. phagocytophilum may also be applicable to other *Rickettsiales*. Our findings are of particular significance, as they provide the first evidence for surface exposure of the intriguing C-terminal repeat extensions of VirB6-3 and VirB6-4. The characterization of a mutant with an insertion into the *virB6-4* gene offers insights about the key role that these T4SS components play in infection of mammalian and tick cells and will facilitate more detailed analysis of the T4SS structural components and the effectors they transport.

## MATERIALS AND METHODS

### Cultivation of Anaplasma phagocytophilum.

The A. phagocytophilum strains used for this work were the wt HZ (58), wt HGE1 (39), and the HGE1 *virB6-4*::Himar1 mutant and *120879*::Himar1 mutant (used for trypsin digestion to confirm cell surface proteins). Two cell lines were used to propagate these bacteria, i.e., HL-60 human promyelocytic cells (ATCC CRL-240) and tick ISE6 cells (ATCC CRL-11974) derived from embryonated eggs of the black-legged tick, Ixodes scapularis. Uninfected and wt or mutant A. phagocytophilum-infected HL-60 cells were maintained in RPMI 1640 medium (Thermo Fisher Scientific) supplemented with 10% heat-inactivated fetal bovine serum (FBS; BenchMark, Gemini Bio-Products), 2 mM l-glutamine (Life Technologies), 0.25% NaHCO_3_ (Sigma-Aldrich), and 25 mM HEPES (Sigma-Aldrich) and were kept at 37°C in a 5% carbon dioxide (CO_2_) atmosphere. Uninfected ISE6 cell cultures were maintained in L-15B300 medium prepared as described previously (59) and supplemented with 5% FBS, 5% tryptose phosphate broth (TPB; Difco, Becton, Dickinson), and 0.1% bovine lipoprotein concentrate (LPC; MP-Biomedical). Infected ISE6 cells were maintained in L-15B300 medium additionally containing 0.25% NaHCO_3_ and 25 mM HEPES buffer.

### RNA extraction and reverse transcription-PCR (RT-PCR).

Total RNA was isolated from infected HL-60 and ISE6 cells using the RNeasy kit (Qiagen) with an added “on-column” DNase I treatment (Qiagen) as per the manufacturer’s instructions and (1 μg) from each sample converted to cDNA by random hexamer priming using the Transcriptor first-strand cDNA synthesis kit (Roche Diagnostics) as per the manufacturer’s instructions. Fluorescence-based RNA concentrations were determined by using the Qubit RNA HS assay kit (Thermo Fisher Scientific) with a Qubit fluorometer (Thermo Fisher Scientific). Specific primers (Table 2) were designed to amplify transcripts from each gene component of the *sodB-virB* operon or intergenic regions. All reactions included primers targeting transcripts from the *msp5* and 16S rRNA genes as internal controls and to ensure integrity of RNA/cDNA. Each reaction contained 200 ng of cDNA combined with 200 μM deoxynucleoside triphosphates (dNTPs), 0.25 μM forward and reverse primers, and 1.25 U of PrimeSTAR GXL DNA polymerase (TaKaRa). PCR amplification conditions were as follows: 94°C for 2 min, 30 cycles of denaturation 98°C for 10 s, annealing at 55°C for 15 s, and extension at 68°C for 1 min, and a final extension step at 68°C for 7 min. PCR products were electrophoretically separated using a 2% SeaKem LE (Lonza) agarose gel, and stained with SYBR gold nucleic acid gel stain (Thermo Fisher Scientific) for UV visualization. Genomic DNA and samples of reactions with no reverse transcriptase for each target were used as positive and negative controls, respectively.

**TABLE 2:** Oligonucleotides used in this study

Purpose and oligonucleotide	Sequence	Amplicon size (bp)	Target
RT-PCR			
AB1727	TGGCAATCCATGAAGCCGAA	230	*sodB*
AB1728	GGAAGCTGCCCCTGAGTAAG		
AB1729	TTAACCAGGCCCACCATGCT	130	*virB3*
AB1730	CGTGCATACCTGGCGCTAAA		
AB1731	AAGCTAGGTTGGTCTTCGGC	347	*virB4-1*
AB1732	CGCCACTGCATATTCACAGC		
AB1733	CCAGAGATCGGGTGTCAGAT	414	*virB6-1*
AB1734	AAGCTCCCCTGCACTTTGTA		
AB1735	TTTGCAGGGCCTATAGGTTG	588	*virB6-2*
AB1736	TGCTGTCGTACCCACTTCAG		
AB1737	TACATAGCTCCGGGTTCTGG	512	*virB6-3*
AB1738	TTCCCAGGTAACGCAGTAGG		
AB1739	TGTCTAGGACTGTCGCATCG	471	*virB6-4* 5′ end
AB1740	TGCGTAAGTTCTGCATCACC		
AB1741	AATCCGGCAAAGAAGAGGAT	132	*virB6-4* 3′ end
AB1742	GTGCCCGAAAACAGCTCTAA		
AB1745	TGTTCGGCTTTTCTTCATGC	204	*msp5*
AB1746	CTTCCTCATCCCCAGTCAGC		
AB1747	AAGAAGTCCCGGCAAACTCC	342	16S
AB1748	CCCACATTCAGCACTCATCG		
AB1727	Same as above	633	*sodB-virB3*
AB1730	Same as above		
AB1729	Same as above	608	*virB3–virB4-1*
AB1732	Same as above		
AB1960	CGAGACCGTTGCCATACTTC	367	*virB4-1–virB6-1*
AB1961	AAACCGAGAGGCCTAAATCC		
AB1962	TCCTCTGATAGTGGAGGTACAG	369	*virB6-1–virB6-2*
AB1963	TAGAGCTATCCTTCTGTCCCTTC		
AB1964	TGGAACAGCATCAGCATCAG	357	*virB6-2–virB6-3*
AB1965	TCCAGAATGCACCCAATACG		
AB1966	TGCCGTGAGGGATGATTTG	362	*virB6-3–virB6-4*
AB1967	CCCTCCACCTTTACTACTATCTC		
RT-qPCR			
AB2053	CGGTAGTGTGGAAGGGTTTAAT	126	*sodB*
AB2054	GCATTCGCTGTGCTAACAAC		
AB2057	GCCGCTGATGGAAGTAAGAT	130	*virB6-1*
AB2058	CCACAACCACCGACGTTATAG		
AB2059	TAGGGTTACCTGGACCGATATT	141	*virB6-2*
AB2060	CTGTCGTACCCACTTCAGAAAG		
AB2061	GCACGTACTGGTGAGGATATT	124	*virB6-3*
AB2062	GTAACGCAGTAGGAGGTGTATC		
AB2063	CTCACACACAGAGGCAGATTT	108	*virB6-4* 5′ end
AB2064	AGAGCATCCAATGGCGAATAG		
AB1741	Same as above	132	*virB6-4* 3′ end
AB1742	Same as above		
AB2065	TGCGGAACTTGGTATGGTATC	118	*msp5*
AB2066	CTCATTTAACCTTTCAACAGTGTCA		
AB2121	AGGGAGGTAGTACGCATCCTAGA	102	*groEL*
AB2122	TGTGATCTCTGGCGACCCATAA		
AB2125	GGCCTATGGTGCTGCTTATAC	115	*rpoB*
AB2126	CCACACTCGAAGTTGCTATCC		
qPCR			
AB1334	AGATGCTGACTGGGGATGAG	125	*msp5* (59)
AB1335	TCGGCATCAACCAAGTACAA		
AB1336^*a*^	CGTAGGTGAGTCTGATAGTGAAGG		
AB2039	GTCAAGTCCGGCACAATCT	111	*crt*
AB2040	CATCTTCTTCTCGGCATCCTT		
AB2041^*b*^	TTGCTGACTGACGACGAAGAGTATGC		
AB2042	CCCAGTCTTGGACTCAGAATTAG	100	*tlr9*
AB2043	GGTATAGCCAGGGATTGGTTAAG		
AB2044^*b*^	TCTAGGTCTCAGTCCTGGTTCTGAAGC		

a Probe labeled with hexachloro-fluorescein (HEX) at the 5′ end and tetramethylrhodamine (TAMRA) at the 3′ end.

b Probe labeled with 6-carboxyfluorescein (6-FAM) at the 5′ end and black hole quencher-1 (BHQ-1) at the 3′ end.

### RT-qPCR.

Transcript differences between *sodB*, *virB6-1*, *virB6-2*, *virb6-3*, and the *virB6-4* 5′ and 3′ ends in the A. phagocytophilum *virB6-4*::Himar1 mutant relative to the wild type were determined using RT-qPCR, and the results were based on the means of three biological replicates (individual RNA extracts). For SYBR green quantitative PCR, cDNA obtained from HL-60 cells infected with the A. phagocytophilum wt or the *virB6-4*::Himar1 mutant was used with primers (Table 2) designed to amplify *sodB*, *virB6-1*, *virB6-2*, *virb6-3*, the *virB6-4* 5′ or 3′ end, *msp5*, *groEL*, and *rpoB* sequences. Reactions were performed using the hot-start LightCycler-FastStart DNA Master SYBR Green I (Roche Diagnostics) in a LightCycler 96 instrument (Roche Diagnostics). Twenty microliters of reaction mixture contained 1× LightCycler FastStart DNA Master SYBR green I, 0.4 μM primers, and 2 μl of template (∼20 ng of cDNA). No-template and no-reverse transcriptase control samples to assess DNA contamination were included for all reactions. The amplification program included the following conditions: 95°C for 10 min and 45 cycles of 95°C for 10 s and 60°C for 30 s. After amplification, a melting curve was acquired by heating of the product at 4.4°C/s to 95°C for 10 s, cooling it at 2.2°C/s to 65°C for 60 s, and then slowly heating at 0.1°C/s to 97°C. After melting-curve acquisition, reaction mixtures were cooled at 37°C for 30 s. Significant differences between the A. phagocytophilum *virB6-4*::Himar1 mutant versus the wt were calculated using Student’s *t* test (*P < *0.05), comparing threshold cycle (Δ*C_T_*) values (target gene − reference gene) of the *virB6-4*::Himar1 mutant and the wild type and calculated based on the Pfaffl formula (60), which yields the gene expression ratio. The expression ratio was then expressed as percent expression by multiplying the gene expression values by 100. The geometric means of Δ*C_T_* values from the reference genes *rpoB* and *groEL* were used for gene expression normalization. Transcript amplification efficiencies for each target were 87% for *sodB*, 99% for *virB6-1*, 97% for *virB6-2*, 100% for *virB6-3*, 96% for the *virB6-4* 5′ end, 100% for *virB6-4*, 93% for *msp5*, 100% for *rpoB*, and 91% for *groEL*.

### VirB6 synthetic peptides and antibodies.

Specific antibodies were raised against A. phagocytophilum strain HZ VirB6 paralogs using synthetic peptides derived from predicted antigenic epitopes using the protein analysis and peptide prediction programs TMpred (61), Phobius (62), BcePred (B-Cell Epitope Prediction of Continuous B-Cell Epitopes in Antigenic Sequences Using Physicochemical Properties) (63), LEPS (Linear Epitope Based on Propensity Scale) (64), and the antigenicity predicting tools from EMBOSS (65). Peptide sequences (56–70 residues) (Table 1) were selected on the basis of their predicted hydrophilicity, antigenic propensity, flexibility, surface probability and lack of sequence identity with other A. phagocytophilum proteins based on BLASTP alignments (66).

VirB6-1 through VirB6-4 peptides were synthesized commercially by LifeTein, NJ. An amino-terminal cysteine was included for conjugation, and 4 mg of each peptide was prepared (2 mg was conjugated to keyhole limpet hemocyanin (KLH) and 2 mg to ovalbumin). Production of polyclonal antibodies against VirB6-1, VirB6-2, VirB6-3, and VirB6-4 was done by Pocono Rabbit Farm and Laboratory Inc., PA. Each rabbit was subcutaneously injected with 200 μg of KLH-conjugated peptide emulsified in complete Freund’s adjuvant. At days 14 and 28 postimmunization, the rabbits were boosted by subcutaneous injection of 200 μg of peptide conjugates in incomplete Freund’s adjuvant. Affinity-purified anti-VirB6 antibodies were prepared by immobilization of sulfhydryl-containing peptides using a commercially available coupling resin (SulfoLink coupling resin; Thermo Fisher Scientific) according to the manufacturer instructions (67). All animal work was performed in accordance with the standards and approval of the University of Florida Institutional Animal Care and Use Committee.

To determine titers and the specificity of the sera for the VirB6 paralogs, we used an enzyme-linked immunosorbent assay (ELISA) in an antibody capture format. For this, 96-well MaxiSorp microtiter plates (Nunc) were coated with 1 μg/well of ovalbumin-conjugated peptide or 2 μg/well of VirB6-ovalbumin-conjugated peptide. Pre- and postimmune sera from each rabbit were serially diluted 10-fold 10^2^ to 10^5^. All assays were performed in triplicate, and antibody binding was detected with recombinant protein A/G conjugated to alkaline phosphatase. Color was developed using 4-nitrophenol phosphate, and the optical densities were measured using an ELISA plate reader at 405 nm.

### Detection of VirB6 paralogs by immunofluorescence.

Antigen slides were prepared from A. phagocytophilum-infected HL-60 and ISE6 cells essentially as described previously (68). Briefly, infected cells were washed twice in 1× phosphate-buffered saline (PBS), collected by centrifugation at 200 × *g* for 5 min at room temperature, fixed in 1 ml of 4% paraformaldehyde–0.0075% glutaraldehyde in PBS for 30 min at room temperature, then washed in PBS, and permeabilized in ice-cold 0.3% Nonidet P-40 (NP-40) for 10 min. After being washed with 1× PBS, the samples were blocked with 1 ml of blocking buffer (5% bovine serum albumin [BSA] and 1% normal goat serum in PBS) and incubated for 3 h at room temperature. After blocking, cells were aliquoted into five 1.5-ml centrifuge tubes and collected by centrifugation at 200 × *g* for 5 min at room temperature. Dual A. phagocytophilum staining was done with a mixture of two primary antibodies, mouse A. phagocytophilum-positive serum (1:320) and the appropriate anti-VirB6 serum, unpurified anti-VirB6-1 serum (1:640), unpurified anti-VirB6-2 serum (1:640), affinity-purified anti-VirB6-3 and anti-VirB6-4 antibodies at concentrations of 0.208 mg/ml and 0.232 mg/ml, respectively, or preimmune rabbit serum (1:640) or protein A-purified 0.768-mg/ml preimmune serum (negative control). All samples were incubated overnight at 4°C. After incubation with primary antibodies, the samples were collected by centrifugation as described above and washed 3 times with washing buffer (1% BSA and 0.1% Tween 20 in PBS), followed by incubation for 1.5 h at room temperature with goat anti-rabbit IgG Alexa Fluor 568-conjugated antibody (Thermo Fisher Scientific) and goat anti-mouse IgG Alexa Fluor 488-conjugated antibody (Thermo Fisher Scientific), both at a dilution of 1:800. The samples then were washed as described above, followed by a final wash with 1× PBS, and mounted with ProLong gold antifade reagent with 4′,6-diamidino-2-phenylindole dihydrochloride (DAPI; Thermo Fisher Scientific).

### Immunoblotting.

A. phagocytophilum organisms were purified from infected HL-60 cells as described previously (69). Host cell free bacterial samples used for immunoblots were resuspended in 1× protein stabilizing cocktail (Thermo Fisher Scientific) and stored at −80°C.

To determine the expression of VirB6-3 and VirB6-4 proteins in wt A. phagocytophilum or the *virB6-4*::Himar1 mutant, host cell purified bacteria were solubilized with *n*-dodecyl-d-maltoside (DDM) lysis buffer (DDM at 1.50%, 1× cOmplete proteinase inhibitor [Roche Diagnostics], MgCl_2_ at 2 mM, NaCl at 150 mM, Tris at 25 mM, and lysozyme at 1 mg/ml) for 20 min at 4°C on a rotator, followed by sonication as described previously (60) using a Q-125A-110 sonicator (Qsonica, Newtown, CT). After sonication, lysates were centrifuged for 10 min at 12,000 × *g* and 4°C. Total protein concentration was determined using the noninterfering protein assay (NI protein assay) kit (G-Bioscience) as per the manufacturer’s instructions. Equal amounts of proteins were loaded per lane and separated on NuPAGE 3 to 8% Tris acetate gels (Thermo Fisher Scientific) and then transferred to polyvinylidene difluoride (PVDF) membranes for immunoblotting. Membranes were cut into strips and probed with either preimmune or postimmunization rabbit anti-VirB6-3, anti-VirB6-4, or A. phagocytophilum-infected dog serum diluted 1:10^3^ fold, followed by incubation with 10 ng/ml of horseradish peroxidase (HRP)-conjugated goat anti-rabbit (SeraCare) or HRP-conjugated anti-dog (1:150,000) antibodies. Antibody reactions against VirB6-3, VirB6-4, or A. phagocytophilum MSP2/p44 were visualized with the chemiluminescent substrate SuperSignal West Femto (Thermo Fisher Scientific).

### Surface trypsin digestion of intact A. phagocytophilum.

Freshly collected A. phagocytophilum strain HGE1 *120879*::Himar1 mutant bacteria, released from HL60 cells, were subjected to trypsinolysis (70), suspended in PBS, and split into aliquots. Sequencing-grade modified trypsin (Promega) and Tris (50 mM; pH 8), at final concentrations of 0.05 mg/ml 1.25 mM, respectively, were added to the trypsin-treated sample. A non-trypsin-treated sample received Tris (50 mM; pH 8) at a final concentration of 1.25 mM. Both samples were incubated at 37°C for 15 min with periodic gentle mixing. After trypsinolysis, the protease inhibitor phenylmethylsulfonyl fluoride (PMSF; 100 mM) (final concentration of 6.25 mM) was added to both samples and incubated for 10 min to inactivate trypsin. After incubation, bacteria from both samples were washed 3 times with 320 μl of wash solution (300 μl of Tris and 20 μl of PMSF) and pelleted by centrifugation at 12,000 × *g* for 10 min at room temperature. Final bacterial pellets were solubilized and proteins resolved as described above. Membranes were cut into strips and screened with anti-VirB6-3, anti-VirB6-4, and anti-mCherry antibodies (1:1,000 mouse anti-mCherry; Novus Biologicals).

### Isolation of *virB6-4*::Himar1 mutant.

A library of mCherry-tagged A. phagocytophilum mutants was generated by Tn mutagenesis using the Himar1 system. The pHimarcisA7mCherry-SS plasmid (49) was introduced into A. phagocytophilum strain HGE1 by electroporation as described previously (40). Following electroporation, bacteria were incubated with HL-60 cells, diluted into three 96-well tissue culture plates, and kept under spectinomycin/streptomycin antibiotic selection (40). Wells containing cells infected with red fluorescent bacteria were expanded into 12-well tissue culture plates. A total of 900 mutants were obtained, and frozen stocks and DNA from each individual culture were archived. Transposon insertion sites were determined by high-throughput genome sequencing of pooled DNA. Briefly, DNA from 25 cultures was combined into one pool, for a total of 36 pools, barcoded, and sequenced on a single lane of an Illumina HiSeq 2000 instrument. Raw Illumina sequencing data were processed and analyzed as described previously (49) using the open-source GALAXY platform located at the University of Florida web site http://galaxy.hpc.ufl.edu. A total of 35,467 reads containing A. phagocytophilum genome-Tn junctions were identified using the A. phagocytophilum strain HGE1 (GenBank accession number APHH01000000) genome and the Himar1 inverted-repeat sequences as references. Through this analysis, a total of 1,200 transposon insertion sites were identified.

Since the DNA used for Tn-seq corresponds to pooled DNA, a PCR screen using primer pairs that bind upstream and downstream of the Tn insertion site within the *virB6-4* gene was used to match this particular mutant to its frozen stock. Reactions used PrimeStar GXL DNA polymerase (TaKaRa) according to the manufacturer instructions, and PCR conditions were as follows: 94°C for 2 min, 30 cycles of denaturation at 98°C for 10 s, annealing at 60°C for 60 s, and extension at 68°C for 3 min, and a final extension step at 72°C for 5 min. PCR products were run on a 1.0% agarose gel and stained with SYBR gold nucleic acid stain. Agarose gel electrophoresis analysis of PCR products showed a PCR product consistent with the size (2,202 bp) of the Tn insertion within the targeted region in the DNA from transformant stock culture number 667 (data not shown). A DNA band of the size of the wild-type locus (368 bp) was also detected in the same stock, indicating the presence of a background mutant population with other Tn insertions. Cloning by limiting dilution resulted in the isolation of a clonal *virB6-4*::Himar1 mutant population.

### Anaplasma phagocytophilum growth curves and cytotoxicity assay.

We compared the growth rates of the A. phagocytophilum *virB6-4*::Himar1 mutant and wild type in HL-60 and ISE6 cells. On the day of infection, 1 ml of uninfected HL-60 cells was seeded in two 24-well tissue culture plates at a density of 3 × 10^5^ cells/ml. A. phagocytophilum wt and *virB6-4*::Himar1 bacteria were purified from heavily infected HL-60 cells (>80% cells contained morulae) and resuspended in 1.2 ml of RPMI 1640. To quantify isolated bacteria, we performed a LIVE/DEAD staining assay (Thermo Fisher Scientific). As the excitation/emission wavelengths from propidium iodide (PI) dye overlap those of mCherry, the PI was replaced with Sytox blue (Thermo Fisher Scientific) to calculate the number of dead cells. Each well was inoculated with host cell-free wt or *virB6-4*::Himar1 organisms at a multiplicity of infection (MOI) of 10 (∼3 × 10^6^ organisms per well) and incubated at 37°C in the presence of CO_2_. After 24 h the inoculum was removed and replaced with 1 ml of fresh RPMI 1640 medium. Triplicate wells of infected cells with each A. phagocytophilum strain were harvested at different time points (1, 2, 3, 4, 5, and 6 days) postinfection (p.i.).

To evaluate growth of the *virB6-4*::Himar1 versus the wild type in ISE6 cell cultures, 2 days before infection, two 24-well tissue culture plates were seeded with 1 ml of uninfected ISE6 cells (3 × 10^5^ cells/ml). On the day of infection, bacteria were purified and resuspended in 1.2 ml of L-15B3SE6 cells. Each well was inoculated with host cell-free wild-type or *virB6-4*::Himar1 bacteria at an MOI of 10. The next day, the medium was replaced and infected cells were collected at different time points (1, 3, 6, 9, and 12 days) p.i. HL-60 and ISE6 cells infected with the A. phagocytophilum wt or *virB6-4*::Himar1 mutant harvested at different times p.i. were processed for DNA extraction, using the Quick-gDNA kit (Zymo Research) as per the manufacturer’s instructions, and the supernatant was used for cytotoxicity assays.

Toxic damage in HL-60 and ISE6 cells due to A. phagocytophilum infection was measured by quantitatively determining the release of lactate dehydrogenase (LDH) into the medium using the Cyto Tox 96 nonradioactive cytotoxicity assay (Promega) following the manufacturer’s instructions. Supernatants from infected cultures were collected at the above-indicated time points p.i. All assays were performed in triplicate, and LDH activity was calculated by measuring optical densities at 490 nm using the Synergy HT plate reader (BioTek Instruments). Final absorbance values were determined after subtracting background values obtained from medium-only or no-cell control wells. To calculate LDH release, supernatants from wells containing uninfected cells (spontaneous LDH activity controls) and from wells containing uninfected cells lysed with 10× lysis buffer (maximum LDH activity controls) were added. Hence, cytotoxicity is expressed as 100× [(LDH activity from infected culture − spontaneous LDH activity controls)/(maximum LDH activity controls − spontaneous LDH activity controls)].

### qPCR.

Duplex quantitative PCR (qPCR) was used to determine A. phagocytophilum GE per host cell as a measure of its growth in cell culture. Quantitation of A. phagocytophilum GE was performed by targeting the single-copy gene *msp5* (Table 2). To normalize bacterial numbers, we used primers and probes targeting the single-copy gene for Toll like receptor 9 (*tlr9*) in HL-60 cells and the calreticulin gene (*crt*) in ISE6 cells. Triplicate reactions with DNA from three experimental wells were used. Reaction mixtures of 20 μl containing 2 μl of genomic DNA, 1× of LightCycler multiplex DNA master mix (Roche Diagnostics), 0.4 μM forward and reverse primers, and 0.2 μM probe were used for amplification in a LightCycler 96 instrument (Roche Diagnostics) with the following conditions: 95°C for 30 s and 45 cycles of 95°C for 5 s and 60°C for 30 s fluorescence detection. To allow for equimolar ratios of templates for standard curve preparation, a linear double-stranded DNA (dsDNA) fragment (Eurofins Genomics) carrying *msp5*, *tlr9*, and *crt* sequences was used. A. phagocytophilum and host cell GE number were calculated based on the standard curve.

### Statistical analysis.

Data are expressed as means ± SEs. Unpaired two-tailed *t* test analysis was used for comparison of growth and cytotoxicity of the A. phagocytophilum wild type versus the *virB6-4*::Himar1 mutant. Statistical comparisons and graphics were made with Prism (GraphPad Software Inc., La Jolla, CA).

## Supplementary Material

Supplemental file 1
