# Supplementary material for: Disruption of VirB6 Paralogs in Anaplasma phagocytophilum Attenuates Its Growth
Source: J Bacteriol. 2020 Nov 4;202(23):e00301-20. doi: 10.1128/JB.00301-20 (PMC7648143; doi:10.1128/JB.00301-20)
Supplement: Supplemental file 1 [file JB.00301-20-s0001.pdf]

Fig. S1A

```
Atumefaciens    1 --------------------------------------------------------------------------------
Ap6-1           1 --------------------------------------------------------------------------------
Ap6-2           1 ----------------------VPRFVPIIFILLFMLAGCGYHGCISQA-NVYEDTVRSVVFANSGEKGQKDSSKYWTKV
Ap6-3           1 -----------------------MLSRSLLFIAIVVLSSCGGT-CIEPGAGVSSSSQEVQVPVYPDGADHKRPVTYWVHS
Ap6-4           1 --------------------------------------------------------------------------------
Am6-1           1 --------------------------------------------------------------------------------
Am6-2           1 MFCNASALQEHVCLVVKWRACVVLRIIPVVF--LMLLSGCGYHGCMSQQ-DAMNDTVRSVVYTNPKQRSD-GTKGQWVKV
Am6-3           1 ------------------------MFRLLLIALVLLISGCGGV-CLEPGKGVSSSSVEVKVPVQPPGASRQNPATYWVNS
Am6-4           1 --------------------------------------------------------------------------------
Ec6-1           1 --------------------------------------------------------------------------------
Ec6-2           1 ------------------------MFKIVLIISIFLLSSCGYHGCIKPQ-SVLFDEYSTTVEA--GLEDGKKEHVAWVKS
Ec6-3           1 -------------------------MKKFIILLLFFVTGCGVN-CIEGSKGISGNSVVVEVSPTVKKVS-KVLQPNWIDT
Ec6-4           1 --------------------------------------------------------------------------------

Atumefaciens    1 --------------------------------------------------------------------------------
Ap6-1           1 --------------------------------------------------------------------------------
Ap6-2          58 DFPLSGKK--LDLQIEPHHLDFCENAQIDV---MELRVANSD------------------------PITMGKNREFKFT-
Ap6-3          57 GYRVGE-KDELKITVD-RTIDLCPLDTKAKPVAIKMYPEH--------------------------FST--PSTEFYD--
Ap6-4           1 --------------------------------------------------------------------------------
Am6-1           1 --------------------------------------------------------------------------------
Am6-2          77 DFPVKDKD--ITLEIEHYNVDFCHDAELSE---LKLEASPGD------------------------DITPGKKKEFTVQ-
Am6-3          56 GYLVKE-DTELKLTVE-NTISLCPKDAS-TPATVKMFPED--------------------------LTSSRSEHEFYD--
Am6-4           1 --------------------------------------------------------------------------------
Ec6-1           1 --------------------------------------------------------------------------------
Ec6-2          54 DLVLAGRQ-FLTIKVDTVNVNFCNNTKDVV----NIFSSEGS------------------------TLD-SSAKKFPVN-
Ec6-3          54 ELRVSKDQPPMRVAVT-GALNMCPKDFLNPKNLIVPAVSCISDIKDISYKDESTYQNIKDVCSNASLRTKAANSEYLSGV
Ec6-4           1 --------------------------------------------------------------------------------

Atumefaciens    1 --------------------------------------------------------------------------------
Ap6-1           1 -----MHRVARALVFLMFLV-VTVPLTSYAAGDATAST----------------PTEEENN---------------LVHY
Ap6-2         108 ----FPVVIMPGEEIRFFLNPVN-NFTID------------------------------DETCRNGKADIRVS-------
Ap6-3         105 ----TYIDVQEGDQLRFSSVLYDLSFPDCKEISREHSPVIFHNNEVIFRDEACKKTVAIEELCLSGATSEVLGDGSKKGL
Ap6-4           1 --------------------------------------------------------------------------------
Am6-1           1 -----MRRAVRAIALM-VLF-VWLPFVPHYAAYSDEPK----------------KEEESDR---------------LVSY
Am6-2         127 ----FPVKIMPKEKIRFSLVPIR-SFTVD------------------------------DAICRNLDEDIYAA-------
Am6-3         105 ----TLLDVVEGDRVTFFPFLYDLEFPSCERIGNEESFVIYYGNSDFYKDKECKLGVNSKDICSSGAYGG----SASLYY
Am6-4           1 --------------------------------------------------------------------------------
Ec6-1           1 -----MLKVSKSIIYVLMLFLINVNFI---CA-KSTLA----------------AEVESEK---------------VAVY
Ec6-2         103 ----IP-NIMKGEKLNFRLSPAL-KFTVD------------------------------KDICKNNNPKVYVQ-------
Ec6-3         133 AHTVSAFRLNKGDAFKVSLIPRKVKVTDCSSAALKELGVIYYSDDEIFEDSNCQQAIPAENICQNGVPKF---------Y
Ec6-4           1 --------------------------------------------------------------------------------

Atumefaciens    1 --------------------------------------------------------------------------------
Ap6-1          44 RHAEATNPRCGAVEELAK-IGSIGITAALGGGVVGVGFLYVPIVGFRPLGFAMIAASIATISAGIAASAPYFACNWSFVR
Ap6-2         146 -----DVGACLSSDI-------GNK--------YPLYFSSK------------------------LGGGSGYYNSWYKVA
Ap6-3         181 LHVKDAKGECKEVPA-------GVQIPRVGGSIIQTPRMQFPLGYIGVVDSS------------VSGELSFSYNNVITN-
Ap6-4           1 --------------------------------------------------------------------------------
Am6-1          43 RYAEATNPRCGAAATVAE-VGAKVIGPSLGLMITGAVLVKIPLGWTTKAGFALIASNVAIISAAVAASSAYFVCNWSFVR
Am6-2         165 -----DGEECKKRDI-------GQQ--------RVINFSHK------------------------LVDKHGFMNAWYEVP
Am6-3         177 KVLKNGKTECEEVRE-------GIELPKLGGSDVQTPRLAFPLGYLSKITRD-----------DKQGVLKSNSKYMIAT-
Am6-4           1 --------------------------------------------------------------------------------
Ec6-1          41 RTTIASNPLYAKVSEHAKYIGIAGFTAGF-ALIIGSIFFLNPATWYVALGMFL--AGMAAIAFAAAKAAPFFACNWSFVR
Ec6-2         140 -----NTSKCMEDYL-------DTE--------YYIPVDNYDFQMNNLMYLK-----------DRGSANKDKEKSWINFP
Ec6-3         204 VK---VNQECKESGGM------AGAIPRLQGTEVSALVDNTDTPYGNKIFYS-----------ED------AKKNWINA-
Ec6-4           1 --------------------------------------------------------------------------------

Atumefaciens    1 --------------------------------------------------------------------------------
Ap6-1         123 HPV------------------------------------------------LRFESADDVSRAAG---GE----------
Ap6-2         182 KGEA--PYITDGIPEYLHISG-GIVKPSSMEKLDDEHEKLLCFNQADLEAHVDKLLKDEK--------------------
Ap6-3         241 ------GRIHDARVPKIN--------------MSPKLDQESCELLKGSNITLRLSNIDSIDQL---------------KD
Ap6-4           1 --------------------------------------------------------------------------------
Am6-1         122 HPV------------------------------------------------LRFEEGETVTGQSG---TPTVLT----EH
Am6-2         201 KNTMASPFVTGFRPHIIPGKSDEASLKKDFPETNKEDQKYLCYSDYDIGNEAKGTNVSQGSAATAVAAKCEVKCLPFGKY
Am6-3         238 ------GRIHDGRTVKIS--------------GPPEITTALCTELKKDKCITRVMEGRSLADL---------------KQ
Am6-4           1 --------------------------------------------------------------------------------
Ec6-1         118 HPI------------------------------------------------MRFDTHDAKRN------------------
Ec6-2         189 LKILYH----LDRKTYRDVSQMNISKLSSDRYQNNNDLFYLCVTDKRLNEYVTKIVEDML--------------------
Ec6-3         257 ------PFVYDARIIKNQ-------------KVNVETLKKHCTIIKELNEKKIINHVDTIS-------------------
Ec6-4           1 --------------------------------------------------------------------------------

Atumefaciens    1 --------------------------------------------------------------------------------
Ap6-1         142 ---------VPTEGDYKECAEP-----------------------------------------------------VAASE
Ap6-2         239 -----------------NSSQDSKEGAKNPAAEKEK----RDKKLAEVRAKVRGVREKFKSSMDAYSLNVHCKKLCSGII
Ap6-3         286 RDAHNKHERSYTYGSNKAFTTGKRFGKPSETEEE-K-----------KQREKEEKIAALLSEYTEYDLNCHCGYVCKPSN
Ap6-4           1 --------------------------------------------------------------------------------
Am6-1         147 TS-NTGDTSSFKIGNYKECENP-----------------------------------------------------HAPDA
Am6-2         281 GYRPGS--KEVPTGCYRVCVDYNKQGAANTATAANT----NDEELNKVLTKVYQARTRYMRAMDELAANEYCGVLKTVQS
Am6-3         283 NQNPGKYEKGV--GCN--------------STQQ-S-----------GGSQTKPDYDALLRGYTEYDISCNCGLICAPSD
Am6-4           1 -------------------------------------MTLLQEK--------SK--------------------------
Ec6-1         132 ---------GNTEGSYKECEEP-----------------------------------------------------TKN--
Ec6-2         245 ----------------------NKTSSGNTAYSGDKKEQLISEKKESLLRKRDAVRKKYQKLYDAYTVNMLCGNLCNIPG
Ec6-3         299 ---TSQ----FEYY-----QKQ--------KAQG-D-----------ITTDIIKELIDVINNFTTYDINCVCGTICNSKS
```

```
Ec6-4          1 --------------------------------------------------------------------------------

Atumefaciens   1 --------------------------------------------------------------------------------
Ap6-1        160 IL---------------------------GK--YGGD------------------CSKEFAYM--ADYYACLAES-----
Ap6-2        298 D-------------------------KSQGCEGYHKL-------LQ-VQGE-EERDVIPFVKLKGNSFALSKL-------
Ap6-3        354 QVD-DDCIRSIVTV-MDGNVVCPTQTKFNGKDHEEDAGNQQHNIPDIRGDLSPEAFARSVSSLTGQHFGQNVAQKDSVIS
Ap6-4          1 -------------------MVVIRF------------------------------TRSNFLRL---LVISCLF-------
Am6-1        173 DVK--------------------AKWESSGTSGGTDG------------------CSKEFADL--NSYFACIAPQ-----
Am6-2        355 STTTTENQSG------------NNSPPKKDYHAYHLL-------SY-DKGNNVLATVLPFVKFGTQTLHPYTF-------
Am6-3        335 QVNKEDCTRSIVRV-MNGKVMCPTTKPKKTIQDNTTI----------DFEQNPDALKDYLD-----------LPADVNDV
Am6-4         10 ----TRNRRS---IGL-GSMVTIAR-----------------------------SAVNKFLR----LLPLCLL-------
Ec6-1        148 ---------------------------------YPDC------------------TNRDFGSE--EEYFRCLVTGDKDST
Ec6-2        303 YDKQKLDKNCFV-V-ETNNMLNSCTIEKSGISGCNL------------NEAGQYSTVLPFLYLTHDSGITNFI-------
Ec6-3        347 KLVTGKEIATISNYGSDGNCIISGSYE-----------------------YNKDATFEEISKAKLEDISKN---------
Ec6-4          1 ----------------------MCN-----------------------------FIKKGFIKL---LLLL-VI-------

Atumefaciens   1 --------------------------------------------------------------------------------
Ap6-1        186 --KSLEDARKAEPTCPSK------KFKKASNYAWPKNRVSSSRYIEVCYRHPLGTVYM-----------------SPYVA
Ap6-2        337 --EKKELPKGME---------KAQVPKEFARDFEDSKLE--------------QAKA---TGTALGQSEEYLVLNRAYEY
Ap6-3        432 VEKAYELAEGVIAVIVSIENGKKILPSDIAQYCND---HQGK-----CKYLPEGIKSLKSSQSGLTFEKGSLKLDSDYIA
Ap6-4         22 --MTSCGNEVPFPRCVSADNFNSSTTIAVS-----------------AYYGSSNVDAF-------KAENGELG---DGSV
Am6-1        208 --KSLEVAKTLEPVCKDR------KFKQVNHYAWPKNRVSSSRYIEVCYRKPLATLFA-----------------GAYRA
Am6-2        408 --KPADSAQ------------KSNESSIFASPDSKQKTESGDTITSQIGNLPTKDDA---VKAGFKDIESYLELDRDYTV
Am6-3        393 KNAAYELAEGAVAVITDK--GK-----DITKDC-----SGSS-----CTFLPEEVKDLSGA---GKFSEQSLRMHKSYEV
Am6-4         42 --VASCGSDVPFPRCVSADNFNSNTTASVS-----------------AYYGPGNVEAF-------KADDGQTG---NAST
Ec6-1        175 KPVSINNAKQNEPVCASQ------KFKKVDKYYWPKNRISSSNYIEVCYRNPIGSLNP-----------------ITMIK
Ec6-2        362 -----------------------------TVDDAVKKWQADNNSSSDIG----------------------LLTNHQYSI
Ec6-3        395 ANNSDPIIQGLTAFFGTRVTHK-QEKDARNYYCFPQKVQGGS-----CYAD------I----SSQNIDSNSLKSNISYAY
Ec6-4         19 --TACESNQHPVPRCVPADVFTETRTASVS-----------------AYFDSRS-EDF-------IADNKSLG---NIV-

Atumefaciens   1 --------------------------------------------------------------------------------
Ap6-1        241 ARLGF--------------------------------AG---------RDP-----------------------------
Ap6-2        389 KTDAAA--------------------------------------------------------RGFILKSSS---------
Ap6-3        504 PGSGRLYLAYWPYFGELGKKVKERKGSEAAVVAASGSSSQRGLTVSRAKNYSENSLLSALAQRRFFWKQGMSSGSGGFLS
Ap6-4         73 PG-SRKQIVRWQDTGLVTDG-------EEIVV------------------------------------------------
Am6-1        263 LKNQT--------------------------------KS---------ESY-----------------------------
Am6-2        471 DEPIK---------------------------------------------------------------IAD---------
Am6-3        453 KKSGRLFLSYIKPKNNSGT-------------------------------------------------------------
Am6-4         93 PG-ANKHMVRWKDTGFVTDG-------EEIVI------------------------------------------------
Ec6-1        232 RASEA--------------------------------SS---------GNV-----------------------------
Ec6-2        391 SKDLDK--------------------------------------------------------------------------
Ec6-3        459 DSD-----------------------------------------------------------------------------
Ec6-4         68 ---KNDQVVRWKYTGYVTDG-------RPIVL------------------------------------------------

Atumefaciens   1 --------------------------------------------------------------------------------
Ap6-1        251 -------------KEVLKESKYPR-------------EATFGDNHMHNTKLAVSGAWT-EFEYTIQCKVLKAGQEAKLHD
Ap6-2        404 -KRAD---------EISLLVEVPK-----------------------EVSGDIAGSYT--LSAKKDCS------------
Ap6-3        584 SNDAGNTYVPLSRDEGITTISYSVASAATPVSAGKPASERRISVARTGEDIAVQGFYS--LNVHRTCY------------
Ap6-4         97 --KVEGAWIPWEKNG----------------EKHSPTMAVNAGIS--RPTDLSDEFYDSVVDVDRICGPYQKYEKQ----
Am6-1        273 -------------TEQIKKGAYPR-------------EGTYGDESMHKTKLAISGAGGDNIGAHVECKVLWAGKEETIHD
Am6-2        479 -SEAH---------TFVLTLEYPE---------N-----GNGTDRRNNTDRILVGSYR--LKVKKDCG------------
Am6-3        472 -----------------------------------------SGRSDTSNKEEMQGFYT--LKVHRTCY------------
Am6-4        117 --SIQGAWVPWAKHG----------------EKHSSPLAVNTGIS--RPVDLNDEFYDSVVDVGRVCGPYEERKHS----
Ec6-1        242 -------------TELLKKGEYPR-------------EGDYSTQEIKVAKYAMQG--------EVKCEVLQAGQEKVIHT
Ec6-2        397 --------------N--LY-----------------------LELRVKDDLELIGKYT--IEVTKDCS------------
Ec6-3        462 --------------ESV-TL-------------------RFAVIGEKQKYASYSGGYN--IHVERICN------------
Ec6-4         90 --KAEGMWTAWVNDV----------------SAEKS---V-------SFGDEDSEHYNQILSVERICGPYNKIEKTFVPD

Atumefaciens   1 --------------------------------------------------------------------------------
Ap6-1        304 ATFRAVERGAKL-CVDA--------VKLSGVPFMAKPEIGCQMR--------PNSPPAPMCAKSVQ--------R-E---
Ap6-2        437 -----AHIGKSL-YYVF--------SE--GPPTFEPGPNN-------------KSRQ-IDFIDTGS-----VF-------
Ap6-3        650 -----ATSGQKL-YMYI--------GD--TPPTALPGKQQ------------GAIPL--DFEKINSSK------S-DKEE
Ap6-4        153 ------VGGCRMQCHAIPM-QDQDGTGQYGRPCWLEKGYGAYLLFKRPNDQDPNATM--DLIEFPESPTTHLAYESAHDG
Am6-1        327 AQFRAVERGDKL-CVDA--------VSVSKLPLIPRPEIGCQMR--------PNSPPVPMCEKSKA-----VM-G-E---
Am6-2        521 -----AHLENSL-YYTF--------SE--SYPEYKPGDSN------------SKSQP-LRFMDKSE-----IR-------
Am6-3        497 -----ATAGKKL-YMYI--------GE--NAPTFLPGKNG------------SNDAVELDLEKDGLNPKGIYYIN-KKKD
Am6-4        173 ------VSGCELNCKHIPA-TDDENTGRYGRPCWLEKGYGAYLLFKRPEDPDPNSTL--DLIEYPVSPTTHLGHAAAGEG
Ec6-1        288 ITFQAVEKADKL-CVYA--------TKSIGIPLWPQVEIGCHMR--------PSGPPVPMCEKSTP-----VM-S-D---
Ec6-2        424 -----NHVKDSL-YYTI--------SK--GPPTVKPGEQG------------T--TP-LNFINNED---TIVK-------
Ec6-3        494 -----FMYGKKL-YMYI--------GD--NPPTIFPGDPG------------TIELFVPDPEN-PESGTGVYMIN-K---
Ec6-4        142 G-----HSECKVSCELITGVKDDLERGAYGPPCWFKNGYGAYLLFKRPDDPEPNETL--DHMRYPVSPVMHIGYKPLELA

Atumefaciens   1 ----------------------------------------------------------MNFTIPA--P-FTAI-------
Ap6-1        355 ------------------TKAADG-SK--------IISYDNSGCYSCYV--AETCKGVANLNSRSIFP-ITSVVVGCIVD
Ap6-2        475 ------------------IDAVGEKGDLYFGIHDNGDGYENNVGYF-----------EIHTKATKIPPRIFGHIVSWTEG
Ap6-3        693 KEW----------SYKINSGAEKRQGYIYFGVDV-DPGYEAKLKQA----NNSDNYYAVHLWVPKWTP-IFSSFFNFLQG
Ap6-4        224 GEAIYR-SSGRIYDVKCQVVNPERGWKVYVKILD-NYYHDNAGGYSL------------EFTKGVVRG-TNTDIFESVRK
Am6-1        380 ------------------AGTERA-GK--------VVKYDNEKCYSCYV--DQACQGTASVHTKSPFP-VTSVLVNCIVG
Am6-2        560 ------------------IEKTSATGDLYYGVRDNGDGESNNIGHF-----------EIKAIISKTPPKFFSHIVKWTEE
Am6-3        548 GNN----------GSNGNNGISGKKGYLYFGIAV-DAEYDNQLKDS----NHPDNYYSVRLWVPTWEP-IISKFFALLQG
Am6-4        244 KEVSYS-SGGKIFGSDCKSIDPQRGWKIYVKILD-NYYYDNAGGYSL------------EFKKGVING-KGVRIFEYVRK
Ec6-1        341 ----------------------DN-KR--------IVSYDNSKCYSCYI--DPACRGEVGPHIKSVFP-MTSIIVSCIKG
```

```
Ec6-2          463 ------------------LTGNDEVGELYFGVKDNGDGYENNTGYF-----------NITVIAKKKIPKVISYMVERLKD
Ec6-3          541 ------------------DGSEKKSGKIYLGIDV-R-GYENQFDYSSLPGIESANKYFVDFFIKRWNP-NFSKVFVMIRD
Ec6-4          215 GSDVFSTKNKPIMNSLCQKVQLEPGWKIYVKILD-KYYYDNVGGYAI------------TFLEGVKTE-KQFSVFEWVRK

                                                                                       TMD1
Atumefaciens    13 --HTIFDVAFTT---------------------------------GLDSML------ETIQEAVSAPLIACVTLWIIVQGI
Ap6-1          405 SLKNLLDPPA----DC----TGRARAMN---------------SVNPGFL------KVAQEKLKKTVMAALILALVLFSI
Ap6-2          526 KVRSALYGSSDT------------------------------------DSVAHKLYRHISQSGPFMRMVNALLVLYVIVSAL
Ap6-3          757 VLLHVLYGTDLPTMGQDTKAVEASKVIGRAMSPEYIGIQGGGQQKKAGVVQQIYNNQVSTKPFWFAVRALLVLYLMFSVL
Ap6-4          289 LVR------------------------------------DELDSAGKKIFQNIAKDKEYRNFVFALITLSIVLTAL
Am6-1          430 SLKNVLDPTSGGGQKC----AHKSTTPG---------------GPNPGFL------KVAQKKLKNAVMAALILALIILFSI
Am6-2          611 RVRSALYGNKDKAGGQ-----------------------------ESVVASMYRHISTDGPFNRLVNALLILYVMVSAL
Am6-3          612 TLLNILYGTEMSTTQGVADVDK-VDNIAS-------AVAKSIKENKKGAIQQIYSNQVTSKPFWMLIQGVLTLYLMFSAL
Am6-4          309 LVR------------------------------------DELDKAGKQIFGNIVENKGFRTFAFAVITLFLVISAL
Ec6-1          387 SLSNILTGQCGQ--------------NA---------------SKKVGFL------KVAQDKLKKAVMAVLVLALIILFAI
Ec6-2          514 SLERGLYGTSSTNSGG---------------------------------VNIIYNSIIENMHFIKIVNSLLVLYILMNAL
Ec6-3          600 FLLRILYGVPKDTQ---------VDNIAQ-------ALDVARTSKSQGAIQSIYANQTGAGSLWRALQALCSLYIVFTVL
Ec6-4          281 QVR------------------------------------GELDKAGEQVFRHLVGNPIFKNFIFSVLTLFLVFGAL

                                         TMD2
Atumefaciens    53 LVIRGEVD-TRSGIT-RVITVTIVVALIV----------------------------GQANYQDYVVSIFEKTVPIFVQQF
Ap6-1          456 KAVLGGVQ-SAGELYMTVIKFALVIYFTQGDAMSLAYEYLTKLSIGLSDIVLRAAGGDTGICDFQASDYSPQYLYLMPWD
Ap6-2          572 YFCFGFSKASIFQLFITIVKIITVMYVLRPDSWTFFNDHLLSIFIDGPKFLIDAMTGGIG-SGGD--------FGFM----
Ap6-3          837 GYIIGIIQVTKHDIFVRIAKIALIITLVSPGSWKFFTEHCFSIFILGIPDIISAFNGYLG-GDSS--------FAFL----
Ap6-4          329 TYIFGMVRAPLTDLIAKLLKIVLVLLLISPGSWDFFYNHLLRLFLEGVDEIIAVVNEYAL-GQKV-FNKNNPFSFM----
Am6-1          485 KAMLGGVQ-NASELYMTAIKFALVVYFTQGDAMSKSYDYLTKLSIGLSDIVLAAANGGQDICNYKTETYDENFRYLVPWD
Am6-2          661 YFFLGFSRASIFQLFIIFAKVIVVYVIRPDSWTFFNDHLFKLFTDGPKSLIAVMTGGVG-NTDD--------FGFM----
Am6-3          684 GYIMGVIKITKYDLAIRVAKVVFLVCVFSQDSWRFFNEHCFSMFIGGVSDIIEAFNGYLD-GDRS--------FKFL----
Am6-4          349 VYLLGMVQAPLADTMVRILKIILVILLVSPGSWDFFHNHLLKLFTGGVDQIIALVNSHAA-GQK--FKSDAPFDFM----
Ec6-1          432 KAALGGIQ-SPAELYMLIIKFALVVYFTQGSAMSHYYDQIVKLSIGLSDMVLQA-GGSQTICDYSQSDYEDKYKYIAPWD
Ec6-2          561 FYCAGFSKASIFELLTITLKISIIIYVIGPNSWDFFNNHLFKLFTEAPIQLISIMTGQDPDTSTS--------FEFL----
Ec6-3          664 GYAIGVVKFTKYDLGVRIAKIAIVVGLISQGSWEFFSDHFFSIFIQGTSDLVAAFNGELD-GDNS--------FRFL----
Ec6-4          321 AYIFGMVQSPFGDIIIRILKISLVILLISPSSWEFFYNHLLALFIHGIDQIIALINSHTG----S-YNPKEPFSFL----

                                                                                             TMD3
Atumefaciens   104 SV-------TGLPLQ--TVPAQLDTIFAVTQAVFQKIAS-EIGPMNDQDIIAFQGAQWVLYGTLWSAFGVYDAVGILTKV
Ap6-1          535 RLDCRMMFYLGSQLTGGTGTGILLTVLLTAGLLIPAILI---NAKVIICLVAFFAVIMLVLTIIWTVYVFL-LSLIALTV
Ap6-2          640 ---------------------DGILYRFSVTQTWIQLLALIFAGPIGWLSVILIFWGIIVLIQFLFQAVVIYLVSIMTIAL
Ap6-3          905 ---------------------DSTLGIMLTSEFWIRMLSLFMAGPVGWLAFIGIIWALFSFFLAMMRAIILYLFIMVGLAF
Ap6-4          403 --DI-----------------MIRDKIFSTVVWEAKARALITADWSSIFALLIIIAVLFYVGLCLYGFVIYLTAFVGVTF
Am6-1          564 RLDCRMMFYLGSQLNGGTGTGVLLTVFLCAGLLIPAILI---NAKVIICVVAFFAVMMLIFTVIWCVYVFL-LSLIALTV
Am6-2          729 ---------------------DGILYRFSVSQTWIQLLALIFSGPVGWITVVLIFWGLFLLVECMFSAVVIYLISIMLIAL
Am6-3          752 ---------------------DATLGILLTSELWIRLLALIAAGPVGWIAFVGVVWALIEFFLAMFEAMIMYLFSILTVAF
Am6-4          422 --DV-----------------MIVDKIFSTVVWEAKARALIMADFASIFALLIIIAVICYVALCLYGFIIYLTAFVGITF
Ec6-1          510 RLDCRVLFYLGQQVNGGAAT-IVLGVLLSAGLLFATMLF---NMKLMICLIAIFAVIMLLIMIWLVYIFL-LSLIALTI
Ec6-2          630 ---------------------DIILYRFSLSESWIQILALLFAGPVGWVSVVLIFWGLVVLFLTIATAVITYLISIILIGL
Ec6-3          732 ---------------------DTTLGILLTGEVWIRLLTLILTGPIGWLIFYIIFWAFFVFFMCVIEAIIAYLLTIVAVAF
Ec6-4          392 --DD----------------MIVNKIFSPVIWKIKIRALIIADFSSIFAVCVIIIAVFIYIALCMYGCVIYLTAFVGITF

                                  *          TMD4
Atumefaciens   174 LLAIGPLILVGYIIFDRTRDIAAKWIGQLITIGLLLLLNLVATIVIL-TE------------------------------
Ap6-1          611 LTIISPLMIPMSLFQATKGFFDGWTRQLMTYSLYPVILFAFLSLMFAVFDNIYFGELKFHRGGSDGTVAGAPKAGQ----
Ap6-2          700 LLCIAPYFLICVLFKRTKSIFDMWIKVLLQTAMQPVLIFSCIALLVHAINEVIYAMLNFEVCDTCVF-------------
Ap6-3          965 LLTLAPIFITFLLFQVTKGLFDGWLKMLVNFMLQPIILFAALAFLNQVIITSLHAVTDFAACESCAV-------------
Ap6-4          465 LISLMPILFVGILFSRFKSLFDGWLTQCISFSMQAILMFTLIAMFGTLIMHYYYRIFGFTACYNEWIHINIP------LI
Am6-1          640 LTIISPLMIPMCLFQVTKGFFDGWTRQLMVYSLYPVILFSFLSLMFTVFDNIYFGELKFQQDDITI------GSSK----
Am6-2          789 LLCIAPFFLICVLFKRTKAIFDAWIKVLLQTAMQPVLIFSCVAVMVQVVNSVIYAMLNFQVCEACVL-------------
Am6-3          812 LITLAPIFFSFILFQRTRQLFDGWLKMLMNFSLQPIIIFASLAFLNQMILTSLHAVTDFTACENCAI-------------
Am6-4          484 LIALMPIMFIGILFSRFRSLFDGWLTQCISFSMQAILMFTLIAMFGSLIMHYYYRIFGFTACYNEVLHIKFYALGH--RI
Ec6-1          585 LILISPLMIPMVLFQATKGFFDGWIRQLMVYTLYPVILFAFLALIFTVFDNIYFEDLKFKRKEVNV------LGQK----
Ec6-2          690 LLSIAPFFIICILFRRTKAIFDSWIKSLVQTAMQPVIIFASFALLTQVIDNIIYAMFNFESCDVCVL-------------
Ec6-3          792 LATLAPIFITFIFFQLTKTLFDAWVKMLVNFSLQPIILFAALAFLNQVVLTVLYKVTSFTVCNQCYL-------------
Ec6-4          454 LIGLFPLFLLGILFSQFKSLFDGWLTQCVSFAMQAILMFTLISLFGALIMNYYYRIFGFTTCYNEWLKVKICVLGKIGCI

                                                                                       TMD5
Atumefaciens   223 ----------------------------------------------------ATALTLM--IGVIT--FAGTTAAKII
Ap6-1          687 -----------------KIWFELVDKK--------------------ACEKRENETNLACI--FDTMQ--FFSRPLVFGI
Ap6-2          767 -------------------NIDL-----------------------------------KLTTLCL--LSF--------PLPIGI
Ap6-3         1032 -------------------GFNISSKD---------------------SKAAP--GQSDICI--IPA--------LLPMGY
Ap6-4          539 INRGYYEWTPGQKYDTITIGKGGTEKR-AYPEAGASARYTFTGGGAVIRVPPDYKDEDFRYVDYPFIDPDTKSKTVPGGV
Am6-1          710 -----------------RVNFKLTNPK--------------------ACDDPKMDSNLACM--FSTVN--FHTKPLVLGL
Am6-2          856 -------------------SLDL-----------------------------------KIATLCV--LRF--------LLPMGI
Am6-3          879 -------------------GLNIPSDD---------------------PGTP--NRPDICL--IPV--------MLPIGF
Am6-4          562 IDQKYYEWTPGQKYDPILIGWSGSSRKGEQTDSGTSARYVFTGGGAIIKVPPDYKDRDFRYVDFPFIDPDAKSSAPPGGV
Ec6-1          655 -----------------RITFTLNDPR--------------------ECDDPKYDNNLACM--FGNIE--FITKPAFFGF
Ec6-2          757 -------------------RPEFDIGI-----------------------------------TTIGFCL--IEF--------LLPIGF
Ec6-3          859 -------------------GFDLPTSA---------------------V-EEG--APYDICL--VSV--------LLPVGY
Ec6-4          534 VDQSIFGWTPGQTYDPKVIGLTTDFNLN-DRKSSGSARYKFTGGGAYIDVPPDRKHKDFRYVDYPFIDPDAKTDGNPFGV

Atumefaciens   245 G-------------------------------------------------------------------------------
Ap6-1          726 SVNA----------------------------------------------------------------------------
Ap6-2          787 IPMI----------------------------------------------------------------------------
Ap6-3         1061 AFEL----------------------------------------------------------------------------
Ap6-4          618 VPRDDTAQGRKFQNLAALVNMLVASERGSSIARIVNKIEKELKQLRSSASVTDGHLSKFEDLVSKKRTE------NPQAN
Am6-1          749 SVTS----------------------------------------------------------------------------
Am6-2          876 VPIM----------------------------------------------------------------------------
Am6-3          907 SNEL----------------------------------------------------------------------------
```

```
Am6-4          642 SPIK-SQDGEKLESLTSLTNSLIASARGTSTARLVEKIKKELEERVKDKTVTSETKDEFIKVIEEREKEDAQGKKDVKQK
Ec6-1          694 NAYA----------------------------------------------------------------------------
Ec6-2          781 SPVS----------------------------------------------------------------------------
Ec6-3          887 SPDL----------------------------------------------------------------------------
Ec6-4          613 TVSQ----DSPFRQLSYYINALLSSNKKYVAARLVKDIEDELDVLEKNGTISLNSKNKVLQIIQQRRTKDNSEKKSELEK

Atumefaciens   246 ----------------------------------------------------LYELDMFFLTGDA---L---IVALPAIA
Ap6-1          730 -------------P------EF-----------KMA-----------TTAQIWTKLGVFVLIGFL---FYHFLGSISYIA
Ap6-2          791 -------------PITDTIREI-----------HNTGE-----AMLIGLPGPIFNILIFLALVHV---GRDFVLNSGEMS
Ap6-3         1065 -------------PVSDRIREG-----------LARGD-----IGFMGLPFSMAMLMVLILACKA---TREFGDIAEVMA
Ap6-4          692 WSDPVFKKQLLDILISEIIANN-AFAGTPLKELEEQYDYSIIKNIKQGWIVMWSEVFGLILIAFLVWQMRAFVQSI---A
Am6-1          753 -------------P------EF-----------KE------------TTAAIWTKLGVFVLVGFL---FYHFLSSISYIA
Am6-2          880 -------------PVSDVVREL-----------YNTGE-----TILIGIPGPLFNVLIFVAVAHA---TRDFVITSGEMC
Am6-3          911 -------------SIDDRHREG-----------LARED-----VGFMGLPFGVAIAMILILCCKS---IRAFVKISETLA
Am6-4          721 YTDLKFKEKLVTTLTTKLIGTSTVLNPTSAKDLEEQHDYNIIKDLKKGYIVMWPEVFGLILVAFLVWQMRAFVQHV---A
Ec6-1          698 -------------P------DF-----------KH------------GAEMLWMKLLMMVLIGFL---FYHFLSSIAYIA
Ec6-2          785 -------------VFTDNVRDS-----------VNSDT-----VMFIGLPMPIVNILIFVIMANA---TKHFVGSSGEMC
Ec6-3          891 -------------SITESIRES-----------YVQGN-----GGLLGLPFDIASALILLLAAHA---MRGFKGMSETIA
Ec6-4          689 YKDEDFKSQIIDSIIADVIGSA-AQERTPQEELNKQYDYWLILRVRAGYLIFWTEVGSLLLAALLIWQMRAFVQSV---A

Atumefaciens   268 GNIGGSY-WSGATQS----ASSL-----------YRRFAQVERG------------------------------------
Ap6-1          766 GELAGDP-RAGVIGS----GGMN-----------PRSIAHKAAGMA----SAVRGAASGK--------------------
Ap6-2          839 SIMFGAF--TNLSEV----GTTA-----------AQS-LLSVVGLD----RQTGQMLEQHQHQSE---------------
Ap6-3         1113 HSISGSM--SGMTAA----AVGA-----------TQS-MLSVVGLD----DATQHLIRSAVAMDP---------------
Ap6-4          768 VALSGGGMMSRTVASMYNEGFARIFSGIPVFGRVIETVDRSIDVARLFARAKIAGIGSAIAEAPEKLIARVPIVGGTIGG
Am6-1          788 GELAGDP-RAGVIGS----GSFN-----------PRALAGGAMGVA----SKISNYASGK--------------------
Am6-2          928 SIMFGAF--TNLSDV----GGSA-----------AQA-LLSVVGMD----KMTGHMAEQQKHQSA---------------
Am6-3          959 HSISGSV--ASITAS----AMGA-----------TQG-MLGVVGLD----AASQQRIAAARGMVP---------------
Am6-4          798 VVLAGGSILSRTVASMYSDGFVRIFSHIPVVGTLIDRVDRGIDGLRVLMRAKAGDLASTATRMPENLLKRVPIVGGVLGT
Ec6-1          733 AELAGDP-RAGHVGS-----SFT-----------PKSMLASIVGLA----AKVQGSVGEQ--------------------
Ec6-2          833 TSIFGSF--ANLSMV----AEGA-----------KES-ALGIIGMD----QNSRMQRSRYQQMNE---------------
Ec6-3          939 HSISGSV--AGLSAS----VYGA-----------TQA-LASIVGRD----QETQNIIKEAMRNRKS--------------
Ec6-4          765 VSLAGGNMMSQTIASMYEGGFMKIFSGIPVVGVVFQKIDQGIDGLKFAV----GNYITAVARMPLNALKSIPYLGAAVK-

Atumefaciens       --------------------------------------------------------------------------------
Ap6-1          806 ------------------------------LSDLGSKARDAI----------KGIGSSDS------------GG------
Ap6-2          882 --------------------LFSRGPGAGRQEGAFSGMMRGF----------GGAHQ--D------------SR------
Ap6-3         1156 -----------VAS--DKVRFDAEDSVQPRHDGVKDNSGADS----------PGK---EG--------------------
Ap6-4          848 MVKGMRHVSGALMSSHTEADLYSMKSISPRFDYARAWLGAHLGYSPLDALQYVGKFSLAKMMGNTSGGLLDNIRQDHAKV
Am6-1          828 ------------------------------MSDAGSKMMDRM----------KGGQAGG-------------GG------
Am6-2          971 --------------------LFYRGAGAGREESFASRLMPTS----------PVAQTGAG------------AP------
Am6-3         1002 -----------PGE--EKLQFENRDGVRPMQEGADPKFSEPD----------GGG---DT------------AA------
Am6-4          878 AAHGVRSLSSALTASHTEEDLYSMRSISPRFDYARAWVGAHLGYSPLDAMKYLGGHALGRITGSETEGLMHNIVQDRAAF
Ec6-1          772 ------------------------------AKKAITDKLDDR-----------KKSASDG------------GG------
Ec6-2          876 --------------------MNNQDSDGERRRDPLDL---HS----------PSR-SDDS------------TN------
Ec6-3          983 -----------TGK--SDVDIVSRSNPNPESRGESV---E----------------------------------------
Ec6-4          840 ---FTAGVTGALTSSYNEYD-RSFSNNFKRLNYARAFIGAHLGFSPLDALKYLGGHVAGKMLGNRDGGLIHNAIEDRKAA

Atumefaciens       --------------------------------------------------------------------------------
Ap6-1          828 -----------TDKVSGGSSG-----------GDSGKGGQS---------------------------------------
Ap6-2          912 -----------GVRRRPVIRP-----------EDPPASF-----------------------------------------
Ap6-3         1190 -----------SDSVQGASNA-----------R----GAGAGEGPGAPVDNDGSNV---GRSGEDN--VSG-GDSGGDIR
Ap6-4          928 MQNLRTLTIGVDKYKPGVYVPKRDEEAD----KNPFRRPDAAEKRK-DE-----------------EDRAKLFDDNGNLH
Am6-1          850 -----------SDKVSGGSSP-----------RSSNAGAGMDSVSGGSSGT-----------------------------
Am6-2         1003 -----------AKRKPIVVHP-----------ADPPASF-----------------------------------------
Am6-3         1038 -----------RAAVQGVDGS-----------TVPDAGPGYDTATGASQDSDSVTGGGAPRVGVPDVTDFG-GPGGGELP
Am6-4          958 LSSLEALTIGVEKYKPSGYAPAQKD-DD----ANPFVRPDLKDGTQGAD-----------------TPSSRLFDSDGKLH
Ec6-1          793 -----------KDELSGGSSE-----------GAGDAAEGAGGGAGGSGAASGGGG------------------------
Ec6-2          904 -----------RTSRGGVSIP-----------ENPPSSFGR---------------------------------------
Ec6-3         1007 --------------RSGGSDS-----------DLPPKGHDGGLRTGISDKG--DNVPQQPQSGDDIKEDSKTGRASEQLH
Ec6-4          916 LDSLKAHILGPEKHRPSPYIPNKKKETEDDDARNPFSKETVGNKSGT--------------------VSPDEYKGGDKNH

Atumefaciens       --------------------------------------------------------------------------------
Ap6-1              --------------------------------------------------------------------------------
Ap6-2              --------------------------------------------------------------------------------
Ap6-3         1238 R-PGGGSSG-----------------------------------------SM--------QAGRPQFEDLLSDDPGINDR
Ap6-4          986 IHRGNFW-----------------------------------------------------------------------DA
Am6-1              --------------------------------------------------------------------------------
Am6-2              --------------------------------------------------------------------------------
Am6-3         1095 L-MGDQSSD-----------------------------------------MV--------Q-GATSGGDEDVSRSGIPDD
Am6-4         1016 VDRENFW-----------------------------------------------------------------------DA
Ec6-1              --------------------------------------------------------------------------------
Ec6-2              --------------------------------------------------------------------------------
Ec6-3         1060 NDVSPLSSS-----------------------------------------SVLDTTPFTLSYNEMQGADNLDSNPSVT--
Ec6-4          976 IIREGLRSEAGSALSDDYKDGDKSHIIREGLRSEAGSALLDGYKGGNGSDIIKGGLPTEGSTLLDRYGNVCVSEGNIEHA

Atumefaciens       --------------------------------------------------------------------------------
Ap6-1              --------------------------------------------------------------------------------
Ap6-2              --------------------------------------------------------------------------------
Ap6-3         1268 VNA------FREYRPGDGGVGEDDRI------SGSTTAGSGVSGG------------------EADVDARVSHSREYRPG
Ap6-4          995 VDAMHGLEVMRGQTNDDAARERIQRDIDRIRDEIVRLSRA-NSSEIPELQDFVMSGGGVD---FDRLRDAALKRRNVDER
Am6-1              --------------------------------------------------------------------------------
```

```
Am6-2              --------------------------------------------------------------------------------
Am6-3         1124 VGVAHYEEVGADAPMGPGGEGAEDDS------YGRDML----SGA------------------SDDEQSGLHTDSQFDAG
Am6-4         1025 VDTLHGLGALRARAEDPEVRDRIDEDMNKLRSEITTVAQE-RPGEVPELAEFVAPSGDVD---FARLMEHSLARHDATRA
Ec6-1              --------------------------------------------------------------------------------
Ec6-2              --------------------------------------------------------------------------------
Ec6-3         1097 ----------RNDTSADGTSGMDSQDA--SSDAGSELHGDGDTGNVATTQDTGDNEGDGAQNGSRDNGENAGNASNVTEQ
Ec6-4         1056 LEVREQLKTMLDNAENDTALQTIQYDCDRLDNALHEYLGD-------------------K---FD---------------

Atumefaciens       --------------------------------------------------------------------------------
Ap6-1              --------------------------------------------------------------------------------
Ap6-2              --------------------------------------------------------------------------------
Ap6-3         1318 DGGVGEGDDRVYGSTTAGS----------GISGDAA-------------GIDARGDHA---REHQPADVGVGEGADRVSG
Ap6-4         1071 DEISGAEAARQGVV-----SQAEERSVADLISGTGESEPSAGGSQALTEERIQGGEGTGDRALPEGIEVGGHELADSASA
Am6-1              --------------------------------------------------------------------------------
Am6-2              --------------------------------------------------------------------------------
Am6-3         1176 PE-WGDEPSLFAGEDVPPA----------GDTGAANSEELRHGAQDS--GAQDSGVHHEDNLDDQSLEQGVGSSDSSDSG
Am6-4         1101 DGTHPAEPGHE---------------------------RGAGGEQGADDEA-----------ARAG----VSEMVDDVLQ
Ec6-1              --------------------------------------------------------------------------------
Ec6-2              --------------------------------------------------------------------------------
Ec6-3         1165 DTSSGENDEVQGGSRDNSESADDASSITEQDSSSGESDEAQGGSRDNSESADDTSSVAEDTSSDESDEAQGGSRDNDENG
Ec6-4         1099 ----------------------------DVTQGYASSRDPMGHPQDLITEDLSKISASSDFRDLNAIDLGTQVQVGSDIG

Atumefaciens       --------------------------------------------------------------------------------
Ap6-1              --------------------------------------------------------------------------------
Ap6-2              --------------------------------------------------------------------------------
Ap6-3         1372 SATA---GSGISGDAVG------------VDARGDHAREHQPADV---GVGE---G----A-----DRVSGSATAG----
Ap6-4         1146 LPENQEGGVD-STAEVVSQA-----EERSVADLISGTG----------ESEPSAGGSQ----ALTEERIQGGEGTGDRAL
Am6-1              --------------------------------------------------------------------------------
Am6-2              --------------------------------------------------------------------------------
Am6-3         1243 A----------DTDEMD------------RADTTSHA-EYSESDI---GQDDQQQG----PEEASADHVQTASSSE----
Am6-4         1139 APDTVDSESGDSMEERSRDT-----ESSALRDAVDEQREVEAGDVDEVQAPPEEEGTSRTPTDRFEDVLSGSEGAATEDL
Ec6-1              --------------------------------------------------------------------------------
Ec6-2              --------------------------------------------------------------------------------
Ec6-3         1245 DSTLSVAEQDTSSDESDEAQGGSRDNDENADDTSSVA-EQDTSSD---ESDEAQGG----SRDNDENADDTSSVAE----
Ec6-4         1151 SDVLQQGSADGNRSEVE-VS-----MDISKRDALLEQME-----------------------------LKGMEAAS----

Atumefaciens       --------------------------------------------------------------------------------
Ap6-1              --------------------------------------------------------------------------------
Ap6-2              --------------------------------------------------------------------------------
Ap6-3         1418 --S-GISGDE-A------GVDARGD---------H---------------------------------------------
Ap6-4         1206 PEGIEVGGHELADSASVSPENQEGGVDS------TAEVVSQAEESSVADLISG-TGESEPSAGGSQALTEERIQGGEGTG
Am6-1              --------------------------------------------------------------------------------
Am6-2              --------------------------------------------------------------------------------
Am6-3         1289 --N-TEQRDDYS------HQASQGY---------Q---------------------------------------------
Am6-4         1214 AGGVVPEGEVGT--RTTAPSDVQGEPRE-------AEP--DPDYDPAPDYDPDPDYDPEPDYDPD---------------
Ec6-1              --------------------------------------------------------------------------------
Ec6-2              --------------------------------------------------------------------------------
Ec6-3         1313 --Q-DTSSDE-S------D-EAQGGSRDNDENDDSASSVTEQDADSV---------------------------------
Ec6-4         1192 --GTE--AQLYDSAELSTPLDVTSSINN------VSSEVSQQDLAS--------NGEIEVSLDKDLELKNLDVVSG----

Atumefaciens       --------------------------------------------------------------------------------
Ap6-1              --------------------------------------------------------------------------------
Ap6-2              --------------------------------------------------------------------------------
Ap6-3         1434 -----------------AREHRL---------------------------------------EDGDTGAGVDNS-DASAD
Ap6-4         1279 DRALPEGIEVGGHELADSASVSPENQEGGVDSTAEVVSQAEESSVADLMSGASDSSSDVSSDTSSDNGDDVNHALKEESS
Am6-1              --------------------------------------------------------------------------------
Am6-2              --------------------------------------------------------------------------------
Am6-3         1306 -----------------AEFVDH---------------------------------------LSDDTGEDTDDSTGGRVG
Am6-4         1268 -PDYDEFFDASQDMLADGEEAAAEPAEG-L--AVEAVSEDEVGTRVTTPSDAQDEPQEVEPRPDYDPDPDYDP-------
Ec6-1              --------------------------------------------------------------------------------
Ec6-2              --------------------------------------------------------------------------------
Ec6-3         1349 ---------------SDGTEVST---------------------------------------SDGDNNLGNDQSTDN---
Ec6-4         1250 -------------DIPDVEVTSSS-----DDVTQIYDSAQDRDSCLDIISSADDASSEVSQQDLASNGE-IEVSLDKDLE

Atumefaciens       --------------------------------------------------------------------------------
Ap6-1              --------------------------------------------------------------------------------
Ap6-2              --------------------------------------------------------------------------------
Ap6-3         1457 VNG-DKVRGAV---------------RD---------------------DLKDGKD------------------------
Ap6-4         1359 V-------TNVGDMEIAEGSGSAFSDPEVRQGGDRS---------------VEVPHAANAAGVRVEDHYHIEDLLSDVGV
Am6-1              --------------------------------------------------------------------------------
Am6-2              --------------------------------------------------------------------------------
Am6-3         1330 IAG-HPVDGAADGYDNGDG----KVIEE---------------------KLNKNPDNDTPA-------------------
Am6-4         1337 ----------TPDY---------DPDPDYDHDPDYD----------------PDPDYDGASGVVQGASA-GDEEAAVEGI
Ec6-1              --------------------------------------------------------------------------------
Ec6-2              --------------------------------------------------------------------------------
Ec6-3         1372 ---------TLDGNENTSGNNDTDVQEE----------------AGKASSVVESSDSDDSD-------------------
Ec6-4         1311 LKNLDVVSGDIPDVEVTSSSDD--VTQVYDSTEDRDSCLDIVSSADDTSSEISQQDLEGNDG------YEID---KDMGS

Atumefaciens       --------------------------------------------------------------------------------
Ap6-1              --------------------------------------------------------------------------------
Ap6-2              --------------------------------------------------------------------------------
Ap6-3         1476 ------------------------------------------------------DK------------------------
```

```
Ap6-4            1417 SDGSLEINFVREESSELGVHEGEGSNYPQKGLSKEKHKGRGTKASSS--------KRD--DKDGAHSGSK--------TD
Am6-1                 --------------------------------------------------------------------------------
Am6-2                 --------------------------------------------------------------------------------
Am6-3            1365 --------------------------------------------------GEKQDKE-----------------------
Am6-4            1381 AAGAMSE----------------GEVDEDRGLSLKKS-DRGRSGTSSPEGGRKSSKKG--YEEDEHDGGEEQLDYGGAHG
Ec6-1                 --------------------------------------------------------------------------------
Ec6-2                 --------------------------------------------------------------------------------
Ec6-3            1408 --------------------------------------------------GRKRGRKDINFLDGPQRKRLYMDEYSSVA-
Ec6-4            1380 SNESMLDEIPGGFA-DVIQDCSDASLSTPLGMSARDDTDA-TDTVEDPKENVLPQDVDLEDLDTTHST-NDIADFSGESS

Atumefaciens          --------------------------------------------------------------------------------
Ap6-1                 --------------------------------------------------------------------------------
Ap6-2                 --------------------------------------------------------------------------------
Ap6-3                 --------------------------------------------------------------------------------
Ap6-4            1479 DDSSGLKASG----GSKEGARFRNKSGKA-PPSG---DRVD--SEAISVGEDALEAIASVPEAEALKTVEIPVDKEGDKR
Am6-1                 --------------------------------------------------------------------------------
Am6-2                 --------------------------------------------------------------------------------
Am6-3                 --------------------------------------------------------------------------------
Am6-4            1442 DDYEGAKESG----LQEVLDKVLS-QGIAAESSA---DLAD--DTAV--SDDMAEQ-------------AAVLHQDTEAQ
Ec6-1                 --------------------------------------------------------------------------------
Ec6-2                 --------------------------------------------------------------------------------
Ec6-3            1437 -------E------------------------------KIAGHESAIAVEEESYER-------------GI-VKKRGK-K
Ec6-4            1457 L-FDATSESDSAAFVGEQSIDFSTEEGSDLPSEVVAQHDVDSQDSSVSVSDMAVADM----------------EQGT--E

Atumefaciens          --------------------------------------------------------------------------------
Ap6-1                 --------------------------------------------------------------------------------
Ap6-2                 --------------------------------------------------------------------------------
Ap6-3                 --------------------------------------------------------------------------------
Ap6-4            1549 DPDEVKAGKKKEEREEIGLIVPNIDQKA-ELEGD----KSEEAPAISGIKQDAVVDSRIIEASRVEGKDYTVEGK-----
Am6-1                 --------------------------------------------------------------------------------
Am6-2                 --------------------------------------------------------------------------------
Am6-3                 --------------------------------------------------------------------------------
Am6-4            1497 DPEEAHTHAAQ----DAGDVEYDVAYSV--AEGEC---AWAEATEMSGYQDDTTTISDEQPEEAVEGATEVAEDRDAEQQ
Ec6-1                 --------------------------------------------------------------------------------
Ec6-2                 --------------------------------------------------------------------------------
Ec6-3            1465 DQQE----------------------------------------------------------------------------
Ec6-4            1518 DSKYTDVDGLSDSVLDLGYLEGNVDNPDIEPEHDMSQQYSTETEELSDMLSDDV-----IEPEGVSETDTQVDSE-ESQD

Atumefaciens          --------------------------------------------------------------------------------
Ap6-1                 --------------------------------------------------------------------------------
Ap6-2                 --------------------------------------------------------------------------------
Ap6-3                 --------------------------------------------------------------------------------
Ap6-4            1619 --PL---AEAASAD--VVTEENVVSAGDEG--LD---------------GTLGAE-VTAGGDVLEATA--SAPEAEALKT
Am6-1                 --------------------------------------------------------------------------------
Am6-2                 --------------------------------------------------------------------------------
Am6-3                 --------------------------------------------------------------------------------
Am6-4            1568 DKGA---TDSDDADDEFEDAVDVAEAGTEADVTL---------------ATLGSHRPEIGGKVASASQ--STGKH-----
Ec6-1                 --------------------------------------------------------------------------------
Ec6-2                 --------------------------------------------------------------------------------
Ec6-3                 --------------------------------------------------------------------------------
Ec6-4            1592 DSGTAGQVEDERVHDEVPYDTQDSTSGDKGDIAEHDDKDGVVEHEDTVDQSIDDT-HEEEGKFTEGIVSESVTEPEGVSE

Atumefaciens          --------------------------------------------------------------------------------
Ap6-1                 --------------------------------------------------------------------------------
Ap6-2                 --------------------------------------------------------------------------------
Ap6-3                 --------------------------------------------------------------------------------
Ap6-4            1672 VEIPVD---KEGDKRDPDEVKAGKKKEEREEIGLIVPNIDQKAELEGDKSEEAPAISGIKQDAVVDSRIIEASRVEGKDY
Am6-1                 --------------------------------------------------------------------------------
Am6-2                 --------------------------------------------------------------------------------
Am6-3                 --------------------------------------------------------------------------------
Am6-4            1623 ----TG---SGGASRR------QQQEERRQMS-------------------------------ALQERIVELQKAL----
Ec6-1                 --------------------------------------------------------------------------------
Ec6-2                 --------------------------------------------------------------------------------
Ec6-3                 --------------------------------------------------------------------------------
Ec6-4            1671 TDTQVDSEESQGDSSASDQV-----EDERV--HDEVPYDT-QDSTSGDK-------------------------------

Atumefaciens          --------------------------------------------------------------------------------
Ap6-1                 --------------------------------------------------------------------------------
Ap6-2                 --------------------------------------------------------------------------------
Ap6-3                 --------------------------------------------------------------------------------
Ap6-4            1749 TVEGKPLAEAASADVVTEENVVSAGDEGLDGTLGAEVTAGGDALEAAASAPEDAALETLDVPVYQEKDGNTATELPREVV
Am6-1                 --------------------------------------------------------------------------------
Am6-2                 --------------------------------------------------------------------------------
Am6-3                 --------------------------------------------------------------------------------
Am6-4            1656 --DSPNVTETDKQSIEAELSTLKAELDGLGGSSQ----------------------------------------------
Ec6-1                 --------------------------------------------------------------------------------
Ec6-2                 --------------------------------------------------------------------------------
Ec6-3                 --------------------------------------------------------------------------------
Ec6-4            1712 ---GDIVEHEDKDSVVEHEGTVDQSIDD-----------------------------------THEEEGKFTEGIVSESV

Atumefaciens          --------------------------------------------------------------------------------
Ap6-1                 --------------------------------------------------------------------------------
```

```
Ap6-2             --------------------------------------------------------------------------------
Ap6-3             --------------------------------------------------------------------------------
Ap6-4        1829 PEAT---EYGTKPDDQEKDGNTATELPREVVPEATEYGTKPDDQEKDGNTATELPREVVPEATEYGTKPDDQDGDKGDLR
Am6-1             --------------------------------------------------------------------------------
Am6-2             --------------------------------------------------------------------------------
Am6-3             --------------------------------------------------------------------------------
Am6-4             --------------------------------------------------------------------------------
Ec6-1             --------------------------------------------------------------------------------
Ec6-2             --------------------------------------------------------------------------------
Ec6-3             --------------------------------------------------------------------------------
Ec6-4        1754 TEPEGVSETDTQVDSEESQGDS--------------------------SASGQVEDERVHDEVPYDTQ-DSTSGDKDDIV

Atumefaciens      --------------------------------------------------------------------------------
Ap6-1             --------------------------------------------------------------------------------
Ap6-2             --------------------------------------------------------------------------------
Ap6-3             --------------------------------------------------------------------------------
Ap6-4        1906 PE--RLDPDIGDGSAIEDEVEVRSSRSSESTDSVPSEVTERDAQRDDQEKDGNTA--TELPREVVPEATEYGTKPDDQ--
Am6-1             --------------------------------------------------------------------------------
Am6-2             --------------------------------------------------------------------------------
Am6-3             --------------------------------------------------------------------------------
Am6-4             --------------------------------------------------------------------------------
Ec6-1             --------------------------------------------------------------------------------
Ec6-2             --------------------------------------------------------------------------------
Ec6-3             --------------------------------------------------------------------------------
Ec6-4        1807 EHEDTVDQSIDDTHEEEG--KFTEGIVSE-SVAEPEGVSETDTQVDSEESQGDSSASGQVEDERVHDEVPYDSQDDTQDS

Atumefaciens      --------------------------------------------------------------------------------
Ap6-1             --------------------------------------------------------------------------------
Ap6-2             --------------------------------------------------------------------------------
Ap6-3             --------------------------------------------------------------------------------
Ap6-4        1980 ----------DGDKGDLRPERLDPDI-GDGSAIEDEVEVRSSRSSE-------STDSVPSEVTERDAQRDDQDGDKGDLR
Am6-1             --------------------------------------------------------------------------------
Am6-2             --------------------------------------------------------------------------------
Am6-3             --------------------------------------------------------------------------------
Am6-4             --------------------------------------------------------------------------------
Ec6-1             --------------------------------------------------------------------------------
Ec6-2             --------------------------------------------------------------------------------
Ec6-3             --------------------------------------------------------------------------------
Ec6-4        1884 TSEDEGDVVEHGDKGDIVAHEDKDSVVEHEGTVDQSIDDTHEEEGKFTEGIVSESVTEPEEVSEADTQVDSEES-Q----

Atumefaciens      --------------------------------------------------------------------------------
Ap6-1             --------------------------------------------------------------------------------
Ap6-2             --------------------------------------------------------------------------------
Ap6-3             --------------------------------------------------------------------------------
Ap6-4        2042 PERLDPDIGDGSAIEDEVEVRSSRSSESTDSVPSEVTERDAQRDDQDGDKGDLRPERLDPDIGDGS-----AIEDEV---
Am6-1             --------------------------------------------------------------------------------
Am6-2             --------------------------------------------------------------------------------
Am6-3             --------------------------------------------------------------------------------
Am6-4             --------------------------------------------------------------------------------
Ec6-1             --------------------------------------------------------------------------------
Ec6-2             --------------------------------------------------------------------------------
Ec6-3             --------------------------------------------------------------------------------
Ec6-4        1959 --------GDSSAS-DQVE-----DERVHDEVPYDI------QDSTSGDKGDIAEHGDKDDIVEHEDTVDQSIDDTHEEE

Atumefaciens      --------------------------------------------------------------------------------
Ap6-1             --------------------------------------------------------------------------------
Ap6-2             --------------------------------------------------------------------------------
Ap6-3             --------------------------------------------------------------------------------
Ap6-4        2114 -EVRSSRSSESTDSVPSEVTERDAQRDDQEKDGNT--ATELPREVVPEATEYGTKPDDQDGDKGDLRPERLDPDIGDGSA
Am6-1             --------------------------------------------------------------------------------
Am6-2             --------------------------------------------------------------------------------
Am6-3             --------------------------------------------------------------------------------
Am6-4             --------------------------------------------------------------------------------
Ec6-1             --------------------------------------------------------------------------------
Ec6-2             --------------------------------------------------------------------------------
Ec6-3             --------------------------------------------------------------------------------
Ec6-4        2019 GKFTEGIV-SESVTEPEEVSEADTQVDSEESQGDSSASDQVEDDKVHDEVPYDSQDDVEHGDKGDIVAHEDKDSV-----

Atumefaciens      --------------------------------------------------------------------------------
Ap6-1             --------------------------------------------------------------------------------
Ap6-2             --------------------------------------------------------------------------------
Ap6-3             --------------------------------------------------------------------------------
Ap6-4        2191 IEDEVEVRSSRSSESTDSVPSEVTERDAQRDDQDGDKGDLRPERLDPDIGDGSAIEDEVEVRSSRSSESTDSVPSEVTER
Am6-1             --------------------------------------------------------------------------------
Am6-2             --------------------------------------------------------------------------------
Am6-3             --------------------------------------------------------------------------------
Am6-4             --------------------------------------------------------------------------------
Ec6-1             --------------------------------------------------------------------------------
Ec6-2             --------------------------------------------------------------------------------
Ec6-3             --------------------------------------------------------------------------------
Ec6-4        2093 VEHEGTV-------------------DQSIDDTHEEEGKFTEGIVSESV----------------------AEPEGVSDV
```

```
Atumefaciens         --------------------------------------------------------------------------------
Ap6-1                --------------------------------------------------------------------------------
Ap6-2                --------------------------------------------------------------------------------
Ap6-3                --------------------------------------------------------------------------------
Ap6-4           2271 DAQRDDQEKDGNTA--TELPREVVPEATEYGTKPDD------------QDGDKGDLRPER--LDPDIGDGSAIEDEVEVR
Am6-1                --------------------------------------------------------------------------------
Am6-2                --------------------------------------------------------------------------------
Am6-3                --------------------------------------------------------------------------------
Am6-4                --------------------------------------------------------------------------------
Ec6-1                --------------------------------------------------------------------------------
Ec6-2                --------------------------------------------------------------------------------
Ec6-3                --------------------------------------------------------------------------------
Ec6-4           2132 DTQVDSEESQGDSSASDQVEDDKVHDEVPYDSQDDTQDSTSEDEGDVVEHGDKGDIVEHEGTVDQSIDDTHEE--EGKFT

Atumefaciens         --------------------------------------------------------------------------------
Ap6-1                --------------------------------------------------------------------------------
Ap6-2                --------------------------------------------------------------------------------
Ap6-3                --------------------------------------------------------------------------------
Ap6-4           2335 SSRSSESTDSVPSEVTERDAQRDDQDGDKGDLRPERLDPDIGDGSAIEDEVEVRSSRSSESTDSVPSEVTERDAQRDDQD
Am6-1                --------------------------------------------------------------------------------
Am6-2                --------------------------------------------------------------------------------
Am6-3                --------------------------------------------------------------------------------
Am6-4                --------------------------------------------------------------------------------
Ec6-1                --------------------------------------------------------------------------------
Ec6-2                --------------------------------------------------------------------------------
Ec6-3                --------------------------------------------------------------------------------
Ec6-4           2210 EGIVS-ESVAEPEEVSEADTQVDSEES-Q------------GDSSAS-DQVED-----ERVHDEVPYD------AQDSTS

Atumefaciens         --------------------------------------------------------------------------------
Ap6-1                --------------------------------------------------------------------------------
Ap6-2                --------------------------------------------------------------------------------
Ap6-3                --------------------------------------------------------------------------------
Ap6-4           2415 GDKGDLRPERLDPDIG----DGSAIEDEVEVRSSR--S----SE-------STDSVPSEVTERDAQRDDQEKDGNTA--T
Am6-1                --------------------------------------------------------------------------------
Am6-2                --------------------------------------------------------------------------------
Am6-3                --------------------------------------------------------------------------------
Am6-4                --------------------------------------------------------------------------------
Ec6-1                --------------------------------------------------------------------------------
Ec6-2                --------------------------------------------------------------------------------
Ec6-3                --------------------------------------------------------------------------------
Ec6-4           2264 GDKGDIAEHGDKDDIVEHDDKGGVVEHEDTVDQSIDDTHEEEGKFTEGIVSESVTEPEGVSETDTQVDSEESQGDSSASD

Atumefaciens         --------------------------------------------------------------------------------
Ap6-1                --------------------------------------------------------------------------------
Ap6-2                --------------------------------------------------------------------------------
Ap6-3                --------------------------------------------------------------------------------
Ap6-4           2476 ELPREVVPEATEYGTKPDDQEKDGNTATELPREVVPEATEYGTKPDDQDGDKGDLRPERLDPD-IGDGS----AIEDEVE
Am6-1                --------------------------------------------------------------------------------
Am6-2                --------------------------------------------------------------------------------
Am6-3                --------------------------------------------------------------------------------
Am6-4                --------------------------------------------------------------------------------
Ec6-1                --------------------------------------------------------------------------------
Ec6-2                --------------------------------------------------------------------------------
Ec6-3                --------------------------------------------------------------------------------
Ec6-4           2344 QVEDERVHDEVPYDSQDDTQDSA----SEDEGDV------------VEHGDKGDIVEHEDKDSVVEHEGTVDQSIDDTYE

Atumefaciens         --------------------------------------------------------------------------------
Ap6-1                --------------------------------------------------------------------------------
Ap6-2                --------------------------------------------------------------------------------
Ap6-3                --------------------------------------------------------------------------------
Ap6-4           2551 ----VRSSRSSESTDSVPSEVTERDAQRDDQEKDGNTA--TELPREVVPEATEYGTKPDDQDGDKGDLRPERLDPDIGDG
Am6-1                --------------------------------------------------------------------------------
Am6-2                --------------------------------------------------------------------------------
Am6-3                --------------------------------------------------------------------------------
Am6-4                --------------------------------------------------------------------------------
Ec6-1                --------------------------------------------------------------------------------
Ec6-2                --------------------------------------------------------------------------------
Ec6-3                --------------------------------------------------------------------------------
Ec6-4           2408 EEGKFTEGIVS-ESVTEPEEVSEADTQVNNEESQGDSSASDQVEDERVHDEVPYDA-QDSTSGDKGDIAEHGDKDDIVEH

Atumefaciens         --------------------------------------------------------------------------------
Ap6-1                --------------------------------------------------------------------------------
Ap6-2                --------------------------------------------------------------------------------
Ap6-3                --------------------------------------------------------------------------------
Ap6-4           2625 SAIEDEVEVRSSRSSESTDSV-PSEVTERDAQRDDQEKDGNTATELPREVVPEATEYGTKPDDQDGDKGDLRPERLDPDI
Am6-1                --------------------------------------------------------------------------------
Am6-2                --------------------------------------------------------------------------------
Am6-3                --------------------------------------------------------------------------------
Am6-4                --------------------------------------------------------------------------------
Ec6-1                --------------------------------------------------------------------------------
Ec6-2                --------------------------------------------------------------------------------
```

```
Ec6-3             --------------------------------------------------------------------------------
Ec6-4        2486 DDKDGVVEH------DDKDGVVEHEDTVDQSIDDTHEEEGKFTEGIVSESVT----------------------------

Atumefaciens      --------------------------------------------------------------------------------
Ap6-1             --------------------------------------------------------------------------------
Ap6-2             --------------------------------------------------------------------------------
Ap6-3             --------------------------------------------------------------------------------
Ap6-4        2704 GDGSAIEDEVEVRSSRSSESTDSVPSEVTERDAQRDDQDGDKGDLRPERLDPDIGDGSAIEDEVEVRSSRSSESTDSVPS
Am6-1             --------------------------------------------------------------------------------
Am6-2             --------------------------------------------------------------------------------
Am6-3             --------------------------------------------------------------------------------
Am6-4             --------------------------------------------------------------------------------
Ec6-1             --------------------------------------------------------------------------------
Ec6-2             --------------------------------------------------------------------------------
Ec6-3             --------------------------------------------------------------------------------
Ec6-4        2532 -----------------------EPEEVSEADTQVDET---LQDLEHEDLKEGADDI--SDSETDENSSGNSD---S---

Atumefaciens      --------------------------------------------------------------------------------
Ap6-1             --------------------------------------------------------------------------------
Ap6-2             --------------------------------------------------------------------------------
Ap6-3             --------------------------------------------------------------------------------
Ap6-4        2784 EVTERDAQRDDQDGDKGDLRPERLDPDIGDGSAIEDEVEVRSSRSSESTDSVPSEVTERDAQRDDQDGDKGDLRPERLDP
Am6-1             --------------------------------------------------------------------------------
Am6-2             --------------------------------------------------------------------------------
Am6-3             --------------------------------------------------------------------------------
Am6-4             --------------------------------------------------------------------------------
Ec6-1             --------------------------------------------------------------------------------
Ec6-2             --------------------------------------------------------------------------------
Ec6-3             --------------------------------------------------------------------------------
Ec6-4        2578 ---------------VSDVEGLASLASIGEESTITQNVKTQSVQSSAI-----LSMKDRDGVLDN----KG-IPS-----

Atumefaciens      --------------------------------------------------------------------------------
Ap6-1             --------------------------------------------------------------------------------
Ap6-2             --------------------------------------------------------------------------------
Ap6-3             --------------------------------------------------------------------------------
Ap6-4        2864 DIGDGSAIEDEVEVRSSRSSESTDSVPSEVTERDAQRDDQEKDGNTATELPREVVPEATEYGTKPDDQEKDGNTATELPR
Am6-1             --------------------------------------------------------------------------------
Am6-2             --------------------------------------------------------------------------------
Am6-3             --------------------------------------------------------------------------------
Am6-4             --------------------------------------------------------------------------------
Ec6-1             --------------------------------------------------------------------------------
Ec6-2             --------------------------------------------------------------------------------
Ec6-3             --------------------------------------------------------------------------------
Ec6-4        2628 ---------DQLDVKDTEKKK-KESSKSKVEN-----SDKKAKSTKATVTPKSLKKT---FKVIL------SQCTQELSN

Atumefaciens      --------------------------------------------------------------------------------
Ap6-1             --------------------------------------------------------------------------------
Ap6-2             --------------------------------------------------------------------------------
Ap6-3             --------------------------------------------------------------------------------
Ap6-4        2944 EVVPEATEYGTKPDDQDGDKGDLRPERLDPDIGDGSAIE--DEVEVRSSRSSESTDSVPSEVTERDAQRDDQEKDGNTAT
Am6-1             --------------------------------------------------------------------------------
Am6-2             --------------------------------------------------------------------------------
Am6-3             --------------------------------------------------------------------------------
Am6-4             --------------------------------------------------------------------------------
Ec6-1             --------------------------------------------------------------------------------
Ec6-2             --------------------------------------------------------------------------------
Ec6-3             --------------------------------------------------------------------------------
Ec6-4        2684 KLSE-----------------AFDKLFIDPQSGKQKRRLSKQDIALMI----EQLKAMIESLKDRKAQVTD---------

Atumefaciens      --------------------------------------------------------------------------------
Ap6-1             --------------------------------------------------------------------------------
Ap6-2             --------------------------------------------------------------------------------
Ap6-3             --------------------------------------------------------------------------------
Ap6-4        3022 ELPREVVPEATEYGTKPDDQDGDKGDLRPERLDPDIGDGSAIEDEVEVRSSRSSESTDSVPSEVTERDAQRDDQEKDGNT
Am6-1             --------------------------------------------------------------------------------
Am6-2             --------------------------------------------------------------------------------
Am6-3             --------------------------------------------------------------------------------
Am6-4             --------------------------------------------------------------------------------
Ec6-1             --------------------------------------------------------------------------------
Ec6-2             --------------------------------------------------------------------------------
Ec6-3             --------------------------------------------------------------------------------
Ec6-4        2734 ----------------PDEM-------------------KAIEESIKQAES-------TIQ-NL----------------

Atumefaciens      --------------------------------------------------------------------------------
Ap6-1             --------------------------------------------------------------------------------
Ap6-2             --------------------------------------------------------------------------------
Ap6-3             --------------------------------------------------------------------------------
Ap6-4        3102 ATELPREVVPEATEYGTKPDDQDGDKGDLRPERLDPDIGDGSAIEDEVEVRSSRSSESTDSVPSEVTERDAQRDDQEKDG
Am6-1             --------------------------------------------------------------------------------
Am6-2             --------------------------------------------------------------------------------
Am6-3             --------------------------------------------------------------------------------
Am6-4             --------------------------------------------------------------------------------
```

```
Ec6-1              --------------------------------------------------------------------------------
Ec6-2              --------------------------------------------------------------------------------
Ec6-3              --------------------------------------------------------------------------------
Ec6-4         2755 ---LNQE-------------------------------------------------------------------------

Atumefaciens       --------------------------------------------------------------------------------
Ap6-1              --------------------------------------------------------------------------------
Ap6-2              --------------------------------------------------------------------------------
Ap6-3              --------------------------------------------------------------------------------
Ap6-4         3182 NTATELPREVVPEATEYGTKPDDQEKDGNTATELPREVVPEATEYGTKPDDQDGDKGDLRPERLDPDIGDGSAIEDEVEV
Am6-1              --------------------------------------------------------------------------------
Am6-2              --------------------------------------------------------------------------------
Am6-3              --------------------------------------------------------------------------------
Am6-4              --------------------------------------------------------------------------------
Ec6-1              --------------------------------------------------------------------------------
Ec6-2              --------------------------------------------------------------------------------
Ec6-3              --------------------------------------------------------------------------------
Ec6-4              --------------------------------------------------------------------------------

Atumefaciens       --------------------------------------------------------------------------------
Ap6-1              --------------------------------------------------------------------------------
Ap6-2              --------------------------------------------------------------------------------
Ap6-3              --------------------------------------------------------------------------------
Ap6-4         3262 RSSRSSESTDSVPSEVTERDAQRDDQEKDGNTATELPREVVPEATEYGTKPDDQDGDKGDLRPERLDPDIGDGSAIEDEV
Am6-1              --------------------------------------------------------------------------------
Am6-2              --------------------------------------------------------------------------------
Am6-3              --------------------------------------------------------------------------------
Am6-4              --------------------------------------------------------------------------------
Ec6-1              --------------------------------------------------------------------------------
Ec6-2              --------------------------------------------------------------------------------
Ec6-3              --------------------------------------------------------------------------------
Ec6-4              --------------------------------------------------------------------------------

Atumefaciens       --------------------------------------------------------------------------------
Ap6-1              --------------------------------------------------------------------------------
Ap6-2              --------------------------------------------------------------------------------
Ap6-3              --------------------------------------------------------------------------------
Ap6-4         3342 EVRSSRSSESTDSVPSEVTERDAQRDDQDGDKGDLRPERLDPDIGDGSAIEDEVEVRSSRSSESTDSVPSEVTERDAQRD
Am6-1              --------------------------------------------------------------------------------
Am6-2              --------------------------------------------------------------------------------
Am6-3              --------------------------------------------------------------------------------
Am6-4              --------------------------------------------------------------------------------
Ec6-1              --------------------------------------------------------------------------------
Ec6-2              --------------------------------------------------------------------------------
Ec6-3              --------------------------------------------------------------------------------
Ec6-4              --------------------------------------------------------------------------------

Atumefaciens       --------------------------------------------------------------------------------
Ap6-1              --------------------------------------------------------------------------------
Ap6-2              --------------------------------------------------------------------------------
Ap6-3              --------------------------------------------------------------------------------
Ap6-4         3422 DQDGDKGDLRPERLDPDIGDGSAIEDEVEVRSSRSSESTDSVPSEVTERDAQRDDQEKDGNTATELPREVVPEATEYGTK
Am6-1              --------------------------------------------------------------------------------
Am6-2              --------------------------------------------------------------------------------
Am6-3              --------------------------------------------------------------------------------
Am6-4              --------------------------------------------------------------------------------
Ec6-1              --------------------------------------------------------------------------------
Ec6-2              --------------------------------------------------------------------------------
Ec6-3              --------------------------------------------------------------------------------
Ec6-4              --------------------------------------------------------------------------------

Atumefaciens       --------------------------------------------------------------------------------
Ap6-1              --------------------------------------------------------------------------------
Ap6-2              --------------------------------------------------------------------------------
Ap6-3              --------------------------------------------------------------------------------
Ap6-4         3502 PDDQDGDKGDLRPERLDPDIGDGSAIEDEVEVRSSRSSESTDSVPSEVTERDAQRDDQDGDKGDLRPERLDPDIGDGSAI
Am6-1              --------------------------------------------------------------------------------
Am6-2              --------------------------------------------------------------------------------
Am6-3              --------------------------------------------------------------------------------
Am6-4              --------------------------------------------------------------------------------
Ec6-1              --------------------------------------------------------------------------------
Ec6-2              --------------------------------------------------------------------------------
Ec6-3              --------------------------------------------------------------------------------
Ec6-4              --------------------------------------------------------------------------------

Atumefaciens       --------------------------------------------------------------------------------
Ap6-1              --------------------------------------------------------------------------------
Ap6-2              --------------------------------------------------------------------------------
Ap6-3              --------------------------------------------------------------------------------
Ap6-4         3582 EDEVEVRSSRSSESTDSVPSEVTERDAQRDDQEKDGNTATELPREVVPEATEYGTKPDDQDGDKGDLRPERLDPDIGDGS
Am6-1              --------------------------------------------------------------------------------
Am6-2              --------------------------------------------------------------------------------
```

```
Am6-3                  --------------------------------------------------------------------------------
Am6-4                  --------------------------------------------------------------------------------
Ec6-1                  --------------------------------------------------------------------------------
Ec6-2                  --------------------------------------------------------------------------------
Ec6-3                  --------------------------------------------------------------------------------
Ec6-4                  --------------------------------------------------------------------------------

Atumefaciens           --------------------------------------------------------------------------------
Ap6-1                  --------------------------------------------------------------------------------
Ap6-2                  --------------------------------------------------------------------------------
Ap6-3                  --------------------------------------------------------------------------------
Ap6-4             3662 AIEDEVEVRSSRSSESTDSVPSEVTERDAQRDDQDGDKGDLRPERLDPDIGDGSAIEDEVEVRSSRSSESTDSVPSEVTE
Am6-1                  --------------------------------------------------------------------------------
Am6-2                  --------------------------------------------------------------------------------
Am6-3                  --------------------------------------------------------------------------------
Am6-4                  --------------------------------------------------------------------------------
Ec6-1                  --------------------------------------------------------------------------------
Ec6-2                  --------------------------------------------------------------------------------
Ec6-3                  --------------------------------------------------------------------------------
Ec6-4                  --------------------------------------------------------------------------------

Atumefaciens           --------------------------------------------------------------------------------
Ap6-1                  --------------------------------------------------------------------------------
Ap6-2                  --------------------------------------------------------------------------------
Ap6-3                  --------------------------------------------------------------------------------
Ap6-4             3742 RDAQRDDQDGDKGDLRPERLDPDIGDGSAIEDEVEVRSSRSSESTDSVPSEVTERDAQRDDQEKDGNTATELPREVVPEA
Am6-1                  --------------------------------------------------------------------------------
Am6-2                  --------------------------------------------------------------------------------
Am6-3                  --------------------------------------------------------------------------------
Am6-4                  --------------------------------------------------------------------------------
Ec6-1                  --------------------------------------------------------------------------------
Ec6-2                  --------------------------------------------------------------------------------
Ec6-3                  --------------------------------------------------------------------------------
Ec6-4                  --------------------------------------------------------------------------------

Atumefaciens           --------------------------------------------------------------------------------
Ap6-1                  --------------------------------------------------------------------------------
Ap6-2                  --------------------------------------------------------------------------------
Ap6-3                  --------------------------------------------------------------------------------
Ap6-4             3822 TEYGTKPDDQEKDGNTATELPREVVPEATEYGTKPDDQDGDKGDLRPERLDPDIGDGSAIEDEVEVRSSRSSESTDSVPS
Am6-1                  --------------------------------------------------------------------------------
Am6-2                  --------------------------------------------------------------------------------
Am6-3                  --------------------------------------------------------------------------------
Am6-4                  --------------------------------------------------------------------------------
Ec6-1                  --------------------------------------------------------------------------------
Ec6-2                  --------------------------------------------------------------------------------
Ec6-3                  --------------------------------------------------------------------------------
Ec6-4                  --------------------------------------------------------------------------------

Atumefaciens           --------------------------------------------------------------------------------
Ap6-1                  --------------------------------------------------------------------------------
Ap6-2                  --------------------------------------------------------------------------------
Ap6-3                  --------------------------------------------------------------------------------
Ap6-4             3902 EVTERDAQRDDQEKDGNTATELPREVVPEATEYGTKPDDQDGDKGDLRPERLDPDIGDGSAIEDEVEVRSSRSSESTDSV
Am6-1                  --------------------------------------------------------------------------------
Am6-2                  --------------------------------------------------------------------------------
Am6-3                  --------------------------------------------------------------------------------
Am6-4                  --------------------------------------------------------------------------------
Ec6-1                  --------------------------------------------------------------------------------
Ec6-2                  --------------------------------------------------------------------------------
Ec6-3                  --------------------------------------------------------------------------------
Ec6-4                  --------------------------------------------------------------------------------

Atumefaciens           --------------------------------------------------------------------------------
Ap6-1                  --------------------------------------------------------------------------------
Ap6-2                  --------------------------------------------------------------------------------
Ap6-3                  --------------------------------------------------------------------------------
Ap6-4             3982 PSEVTERDAQRDDQEKDGNTATELPREVVPEATEYGTKPDDQDGDKGDLRPERLDPDIGDGSAIEDEVEVRSSRSSESTD
Am6-1                  --------------------------------------------------------------------------------
Am6-2                  --------------------------------------------------------------------------------
Am6-3                  --------------------------------------------------------------------------------
Am6-4                  --------------------------------------------------------------------------------
Ec6-1                  --------------------------------------------------------------------------------
Ec6-2                  --------------------------------------------------------------------------------
Ec6-3                  --------------------------------------------------------------------------------
Ec6-4                  --------------------------------------------------------------------------------

Atumefaciens           --------------------------------------------------------------------------------
Ap6-1                  --------------------------------------------------------------------------------
Ap6-2                  --------------------------------------------------------------------------------
Ap6-3                  --------------------------------------------------------------------------------
Ap6-4             4062 SVPSEVTERDAQRDDQDGDKGDLRPERLDPDIGDGSAIEDEVEVRSSKKTLLMRRGQIDVSVVSTATKDIASEALKSGKE
```

```
Am6-1              --------------------------------------------------------------------------------
Am6-2              --------------------------------------------------------------------------------
Am6-3              --------------------------------------------------------------------------------
Am6-4              --------------------------------------------------------------------------------
Ec6-1              --------------------------------------------------------------------------------
Ec6-2              --------------------------------------------------------------------------------
Ec6-3              --------------------------------------------------------------------------------
Ec6-4              --------------------------------------------------------------------------------

Atumefaciens       --------------------------------------------------------------------------------
Ap6-1              --------------------------------------------------------------------------------
Ap6-2              --------------------------------------------------------------------------------
Ap6-3              --------------------------------------------------------------------------------
Ap6-4         4142 EDVVRDTSSKVAAPIEDRGLLKTESDVTTAAQKQSDTDTLELFSGTSSDKKKAKKGDAGKSKKRKPKRKEGQPSGVSTHE
Am6-1              --------------------------------------------------------------------------------
Am6-2              --------------------------------------------------------------------------------
Am6-3              --------------------------------------------------------------------------------
Am6-4              --------------------------------------------------------------------------------
Ec6-1              --------------------------------------------------------------------------------
Ec6-2              --------------------------------------------------------------------------------
Ec6-3              --------------------------------------------------------------------------------
Ec6-4              --------------------------------------------------------------------------------

Atumefaciens       --------------------------------------------------------------------------------
Ap6-1              --------------------------------------------------------------------------------
Ap6-2              --------------------------------------------------------------------------------
Ap6-3              --------------------------------------------------------------------------------
Ap6-4         4222 ILKGLLSTMTSENISHQDKSLPEKDQEQDASKETHQDEKKKSQEKEREEKAQKQKSSISKMQRKISKLKSRIAQGNLTED
Am6-1              --------------------------------------------------------------------------------
Am6-2              --------------------------------------------------------------------------------
Am6-3              --------------------------------------------------------------------------------
Am6-4              --------------------------------------------------------------------------------
Ec6-1              --------------------------------------------------------------------------------
Ec6-2              --------------------------------------------------------------------------------
Ec6-3              --------------------------------------------------------------------------------
Ec6-4              --------------------------------------------------------------------------------

Atumefaciens       ---------------------
Ap6-1              ---------------------
Ap6-2              ---------------------
Ap6-3              ---------------------
Ap6-4         4302 EIQVLKNKIAEIEADINGLDS
Am6-1              ---------------------
Am6-2              ---------------------
Am6-3              ---------------------
Am6-4              ---------------------
Ec6-1              ---------------------
Ec6-2              ---------------------
Ec6-3              ---------------------
Ec6-4              ---------------------
```

Fig. S1B

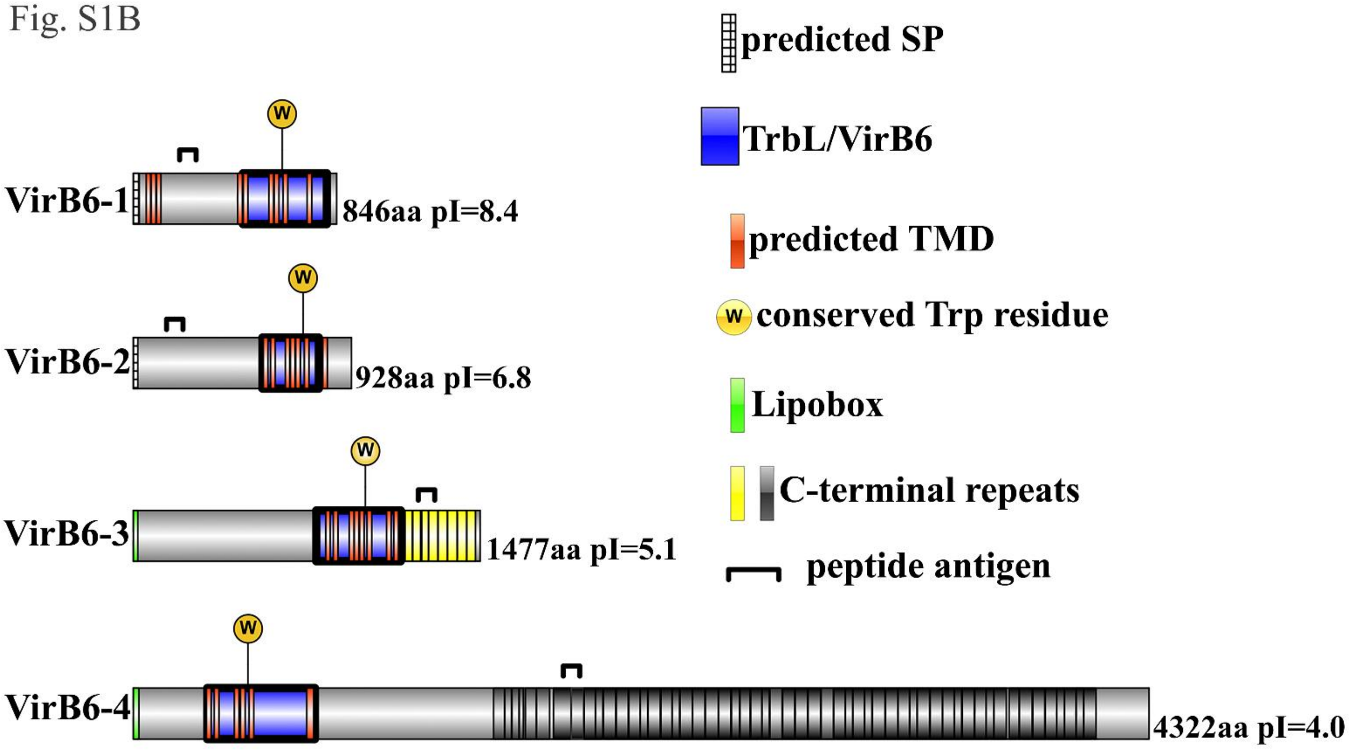

Fig. S1. **VirB6 proteins in *A. phagocytophilum***. **A**. Multiple sequence alignment of VirB6 encoded amino acid sequences. *A. tumefaciens* (Atumefaciens) VirB6 (GenBank accession number P09779) was aligned to orthologs from *A. phagocytophilum* (Ap6-1-6-4) (GenBank accession numbers; WP_011450503, WP_052689204, WP_011450505, WP_080651062), *A. marginale* (Am6-1-6-4) (GenBank accession numbers; WP_011114444, WP_011114443, WP_011114442, WP_011114441) and *E. chaffeensis* (Ec6-1-6-4) (GenBank accession numbers; WP_011452637, WP_006009887, WP_011452638, WP_011452639) using Clustal with default options and linked, for presentation, to BoxShade available at https://embnet.vital-it.ch/cgi-bin/BOX_form_parser.with. Identical amino acid residues are shaded with black background while conservative residues are in grey background. Representative *A. phagocytophilum* str. HZ (GenBank accession), *A. marginale str*. St Maries (GenBank accession) and *E. chaffeensis* str. Arkansas (GenBank accession) were used. The five TMDs of *A. tumefaciens* are underlined in red and identified as TMD1-TMD5. *A. tumefaciens* VirB6 cytoplasmic loop is highlighted in yellow. The invariant W residue located within a cytoplasmic loop is indicated by an asterisk *. **B**. Characteristics of the *A. phagocytophilum* VirB6 proteins. VirB6s size and isoelectric points (pI) are indicated.

Putative signal sequences and TMDs were based on predictions using SignalP v. 4-1, DOLOP for lipoprotein prediction (1) and the transmembrane helix prediction tool TOPCONS (2). The TrbL/VirB6 domain was identified using the NCBI Conserved Domain Database (CCD v. 3.15-4863). Proteins were drawn using the Illustrator for Biological Sequences (IBS) software (3).

Fig. S2

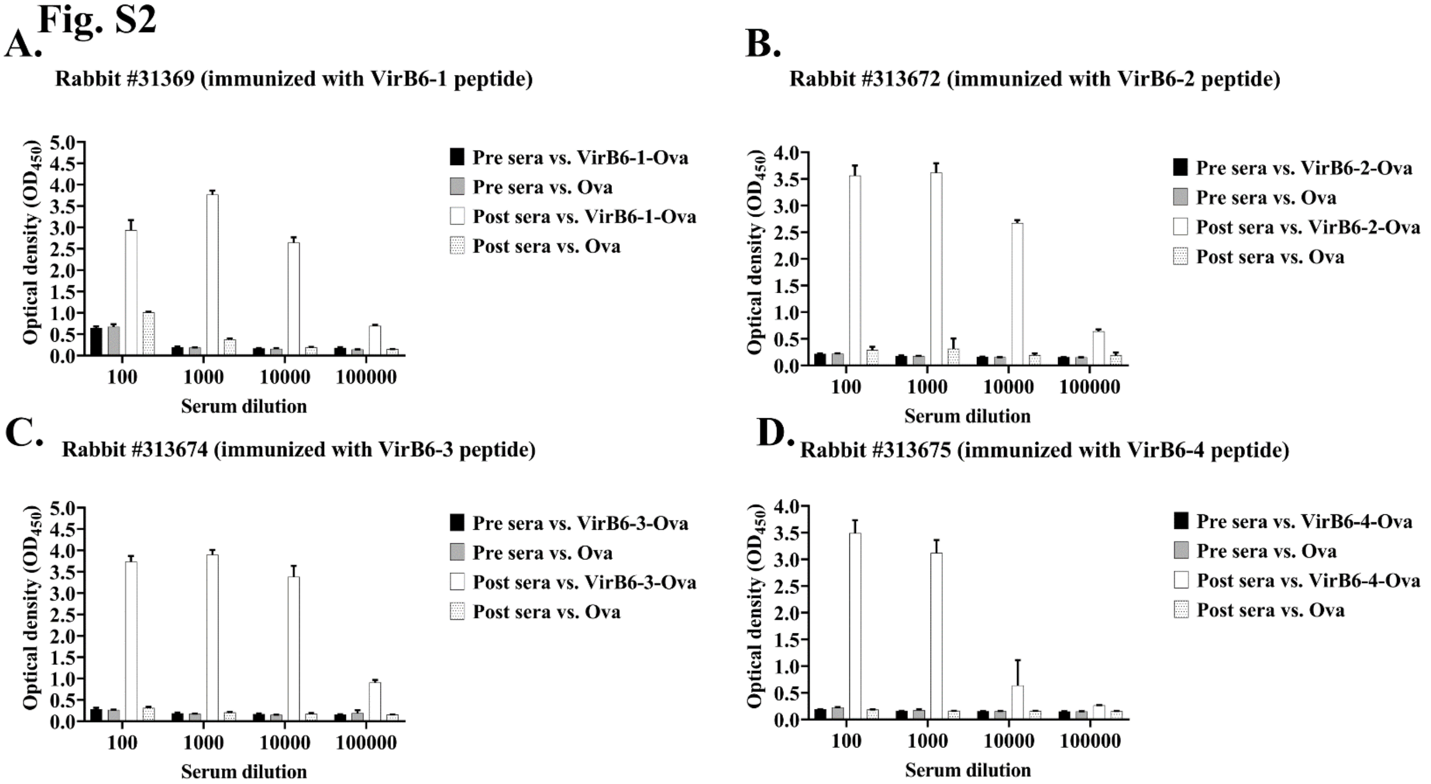

Fig. S2. Immunized rabbits produced antibodies specific to VirB6 peptides. A-D. Antibody responses were measured using VirB6-1, VirB6-2, VirB6-3 and VirB6-4 ovalbumin-conjugated synthetic peptide and ovalbumin as antigens reacted with pre-immunization and post-immunization rabbit serum.

Fig. S3

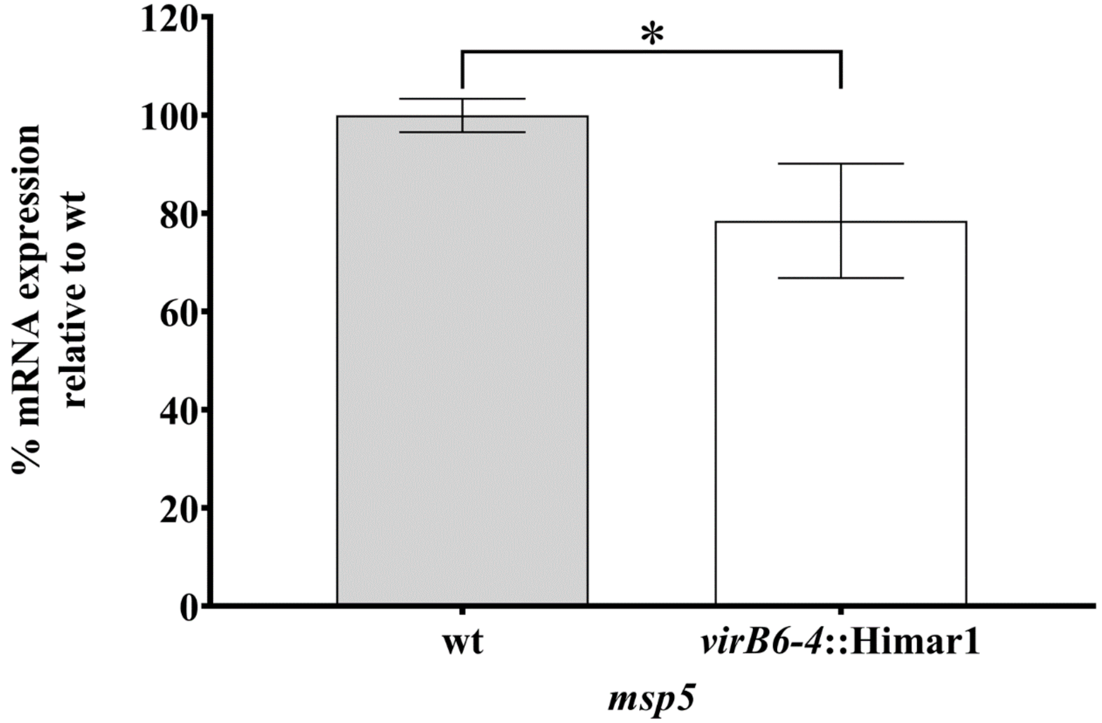

Fig. S3. Deletion of virB6-4 affects the transcription of *msp5*. Changes in expression of this gene was calculated based on geometric mean of the ΔCT values from reference genes *rpoB* and *groEL*, and the results were expressed as percentage of expression, with a 100% expression level being assigned to the control group, in this case, wt *A. phagocytophilum*. Bar lengths represent the percentage of expression of *msp5* in *A. phagocytophilum* (wt) (grey bars) and *virB6-4*::himar1 mutant (white bars). *, P = 0.01 to 0.05.

References

1. Babu MM, Priya ML, Selvan AT, Madera M, Gough J, Aravind L, Sankaran K. 2006. A database of bacterial lipoproteins (DOLOP) with functional assignments to predicted lipoproteins. *J Bacteriol* 188:2761-73. doi: 10.1128/JB.188.8.2761-2773.2006
2. Tsirigos KD, Peters C, Shu N, Kall L, Elofsson A. 2015. The TOPCONS web server for consensus prediction of membrane protein topology and signal peptides. *Nucleic Acids Res* 43:W401-7. doi: 10.1093/nar/gkv485.
3. Liu W, Xie Y, Ma J, Luo X, Nie P, Zuo Z, Lahrmann U, Zhao Q, Zheng Y, Zhao Y, Xue Y, Ren J. 2015. IBS: an illustrator for the presentation and visualization of biological sequences. *Bioinformatics* 31:3359-61. doi: 10.1093/bioinformatics/btv362.
